# Collagenase IV and clusterin-modified polycaprolactone-polyethylene glycol nanoparticles for penetrating dense tumor tissues

**DOI:** 10.7150/thno.47446

**Published:** 2021-01-01

**Authors:** Hao-Yan Huang, Li-Qing Chen, Wei Sun, Huan-Huan Du, Shunli Dong, Atef Mohammed Qasem Ahmed, Dingyun Cao, Jing-Hao Cui, Yi Zhang, Qing-Ri Cao

**Affiliations:** 1 College of Pharmaceutical Sciences, Soochow University, Jiangsu Province, Suzhou 215123, People's Republic of China; 2 Suzhou No3 high school sino-us course center, Jiangsu Province, Suzhou 215001, People's Republic of China

**Keywords:** extracellular matrix, collagenase IV, clusterin, PCL-PEG nanoparticles, doxorubicin

## Abstract

**Purpose:** Novel collagenase IV (ColIV) and clusterin (CLU)-modified polycaprolactone-polyethylene glycol (PCL-PEG) nanoparticles that load doxorubicin (DOX) were designed and fully evaluated *in vitro* and *in vivo*.

**Methods:** PCL-PEG-ColIV was synthesized by linking PCL-PEG and ColIV through a carbodiimide method. DOX-loaded nanoparticles (DOX-PCL-PEG-ColIV) were self-assembly prepared, followed by noncovalently adsorbing CLU on the DOX-PCL-PEG-ColIV surface to obtain DOX-PCL-PEG-ColIV /CLU nanoparticles, which can penetrate through the tumor extracellular matrix (ECM) and inhibit phagocytosis by macrophage. The physicochemical properties of nanoparticles were characterized. The cellular uptake and antiphagocytosis ability of nanoparticles in MCF-7 tumor cells and RAW264.7 cells were investigated. The penetration ability of nanoparticles was individually evaluated in the two-dimensional (2D) and three-dimensional (3D) ECM models. The tissue distribution and antitumor effect of nanoparticles were evaluated in MCF-7 cell-bearing nude mice.

**Results:** Compared with DOX-PCL-PEG-COOH nanoparticles, DOX-PCL-PEG-ColIV/CLU nanoparticles could effectively overcome the phagocytosis by RAW264.7 and showed excellent cellular uptake in MCF-7 cells. In addition, they showed remarkable penetration ability through the 2D and 3D ECM models. DOX-PCL-PEG-ColIV/CLU nanoparticles significantly reduced the drug distribution in the liver and spleen and enhanced the drug accumulation in tumor tissue compared with DOX-PCL-PEG-COOH or DOX-PCL-PEG-ColIV nanoparticles. DOX-PCL-PEG-ColIV/CLU nanoparticles showed remarkable antitumor effect but did not cause severe pathological damages in the main tissues, including the heart, liver, spleen, lung, and kidney.

**Conclusion:** Novel ColIV and CLU-modified PCL-PEG nanoparticles showed excellent cellular uptake, ECM penetration, antiphagocytosis, and antitumor effects both *in vitro* and *in vivo*.

## Introduction

Direct application of traditional chemotherapy causes serious damages to the normal tissues of cancer patients 1,2. Numerous nanobased drug delivery systems have been applied in preclinical and clinical trials. However, the current designs of nanocarriers still have some major drawbacks 3,4. One of the challenges in cancer therapy is the tumor microenvironment barrier, which includes hypoxia, acidic pH, intercellular pressure, extracellular matrix (ECM) barrier, and drug-resistant protein 5-7. ECM, which is a three-dimensional (3D) macromolecular network with unique biological characteristics, is one of the major barriers 8,9. ECM components can significantly affect drug delivery. They change significantly during tumorigenesis, thereby leading to increased fibrosis matrix formation, matrix stiffness, excessive deposition of ECM components, and even abnormal ECM remodeling 10-12.

For improving the delivery efficiency of various nanomaterials to the tumor site, remodeling or reconstruction strategies of the ECM might provide novel ideas. Some studies have already demonstrated that ECM remodeling could improve the tumor targeting efficiency of the nanoparticles 13-15. Lee *et al.* developed the ECM remodeling strategy by the pulsed high intensity focused ultrasound technology for enhanced tumor-targeting of nanoparticles 13. Murty *et al.* found that modifying the surface of PEGylated gold nanoparticles with collagenase could improve the accumulation of nanoparticles within a murine tumor xenograft 14. Zinger *et al.* showed that a pretreatment based on a proteolytic-enzyme nanoparticle system disassembles the dense pancreatic ductal adenocarcinoma collagen stroma and increases drug penetration into the pancreatic tumor 15.

In addition, various novel methods have also been introduced for improving the penetration of nanomedicine to tumor cells 16,17. Zhang *et al.* constructed polystyrene nanoparticles with similar sizes and charges, but with different surface topologies at the molecular level, by conjugating poly (propylene imine) dendrimers with different generations onto the nanoparticles. They found that subtle changes made to the surficial chemical properties led to changes in surface roughness and wettability, which considerably influenced the cellular internalization, endocytosis mechanism, and penetration into the tumor model both in vitro and in vivo 16. Meanwhile, scientists have reported a single-step microfluidic combinatorial approach for producing a library of single and dual-ligand liposomes with systematically-varied properties including size, zeta potential, targeting ligand, ligand density, and ligand ratio. The folic acid and TAT peptide dual-ligand liposome demonstrated an enhanced tumor penetration as observed using two-dimentional (2D) cell monolayer models and 3D tumor spheroid models 17.

Collagen is the most abundant component of ECM and forms the ECM scaffold. It has four major types 18. Type I and III collagens mainly exist in connective tissues, such as skin and blood vessels 19. Type II collagen is mainly produced by chondrocytes, mostly in the bones, joints, tendons, and other tissues 20,21. Type IV collagen is an important component of the basement membrane. During tumor growth, fibrous tissue proliferates actively, and a large amount of collagen deposition exists 22,23. All kinds of collagen increase, but the most important increase is that of type Ⅳ collagen, which constitutes the basement membrane. Zhou *et al.* found that hyaluronidase-modified nanoparticles could penetrate tumor ECM, increase drug delivery at the tumor site and repair the penetrated part of ECM immediately without affecting the leakage or metastasis of tumor cells 24. Therefore, to assume that nanocarriers modified with collagenase Ⅳ can help them penetrate tumor ECM and increase the amount of drugs in tumor cells is reasonable.

However, traditional nanocarriers generally have strong nonspecific protein adsorption, easily uptake by macrophages especially for larger particles (>200 nm), and then accumulated in the liver and spleen 25. Recently, it has been found that various 100 nm nanoparticles showed highly significant and specific liver deposition 26. This condition can greatly reduce the amount of nanocarriers reaching the tumor site, with a relatively short systemic circulation time in the body 27,28. Polyethylene glycol (PEG) is widely used to modify drugs or nanocarriers to reduce the protein adsorption and achieve the “stealth” effect 29,30. By engineering a hierarchical PEG structure on nanoparticle surfaces, an alternative mechanism to enhance nanoparticle blood circulation with a half-life varying from 4 to 10 hours has been achieved 31. Hydration of hydrophilic polyether scaffold reduces the protein adsorption on hydrophobic polymer surface by spatial repulsion. It reduces nonspecific recognition by phagocytosis and prolongs the circulation time by reducing the renal clearance *in vivo*. However, PEGylation cannot completely inhibit protein adsorption on the surface of nanocarriers 32. Some proteins, such as serum proteins, are always adsorbed on the surface of nanocarriers. Thus, the surface properties are changed 33.

Recently, some researchers have analyzed the components of protein corona on the surface of PEGylated nanocarriers after treatment with plasma or serum and found that the clusterin (CLU) expression was most abundant. Compared with human serum albumin-modified nanocarriers, which were significantly taken up by the macrophage, the increase of CLU amount could increase the hydrophobicity of nanoparticles and reduce the adsorption of nonspecific proteins, thereby achieving an antiphagocytosis effect 34. As a high density lipoprotein existing in plasma 35, CLU was adsorbed to the hydrophilic surface by hydrogen bond to form CLU hard protein corona *in vivo*. This condition reduced the adsorption of serum albumin 36,37. In addition, as the only *in vivo* receptor of CLU, low density lipoprotein receptor-related protein 2 is a cell surface protein discovered in recent years 38. It belongs to endocytotic receptor family and is widely expressed in various tissues and organs but is less expressed in granulocyte/macrophage lineage cells 39. Therefore, we hypothesize that ColIV and CLU modification of carriers can not only degrade the collagen component of tumor ECM to deliver several carriers but also effectively reduce the nonspecific protein adsorption compared with traditional PEGylated carriers.

**Scheme 1:**
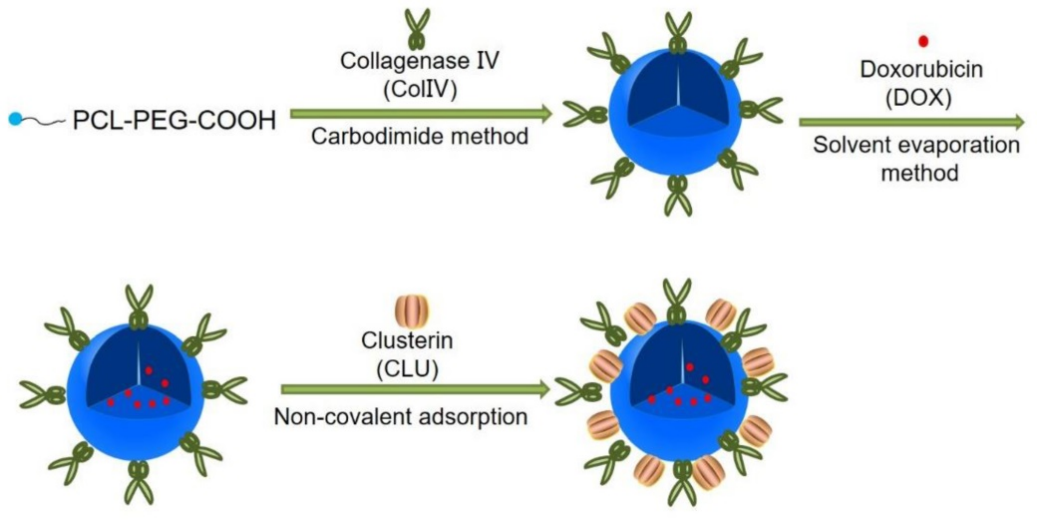
Preparation of DOX-PCL-PEG-ColIV/CLU nanoparticles. PCL-PEG-ColIV was synthesized by linking PCL-PEG and ColIV through a carbodiimide method. DOX-PCL-PEG-ColIV nanoparticles were self-assembly prepared, followed by noncovalently adsorbing CLU on the DOX-PCL-PEG-ColIV surface to obtain DOX-PCL-PEG-ColIV/CLU nanoparticles.

**Scheme 2:**
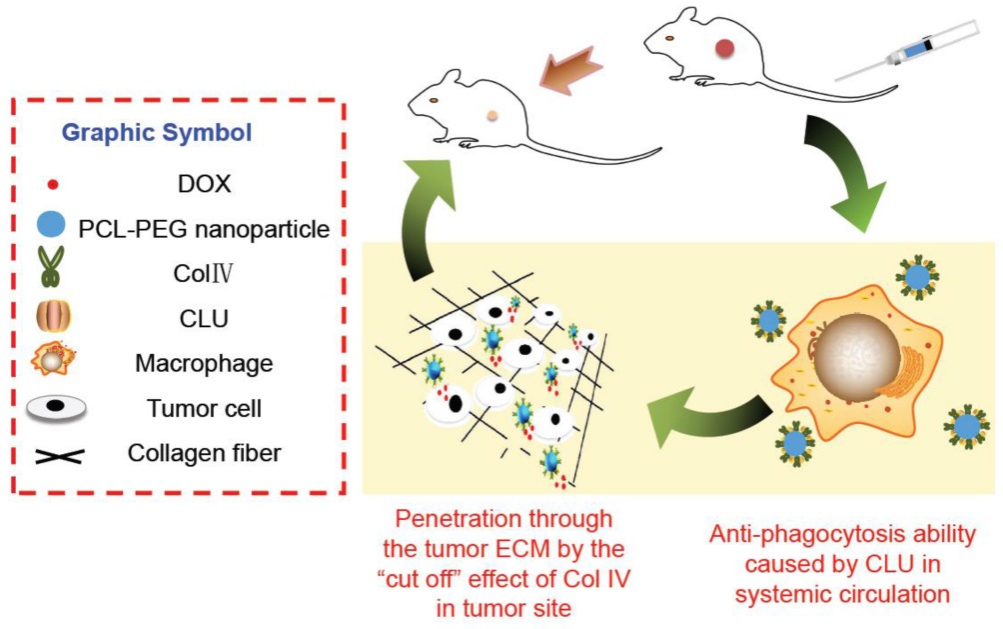
Cellular mechanisms of DOX-PCL-PEG-ColIV/CLU nanoparticles. ColIV covalently linked onto the nanoparticles degrades the collagen component of ECM, thereby resulting in enhanced penetration of nanoparticles through the tumor ECM. CLU can form the protein corona on the surface of nanoparticles and further inhibit phagocytosis by macrophage in the systemic circulation.

In this study, PCL-PEG-ColIV was synthesized by linking PCL-PEG and ColIV through a carbodiimide method. DOX-loaded nanoparticles (DOX-PCL-PEG-ColIV) were self-assembly prepared, followed by noncovalently adsorbing CLU on the DOX-PCL-PEG-ColIV surface to obtain DOX-PCL-PEG-ColIV/CLU nanoparticles, which can penetrate through the ECM and inhibit phagocytosis by macrophage (Schemes 1 and 2). The physicochemical properties of functionalized nanoparticles were characterized in terms of particle size, morphology, drug loading, entrapment efficiency, and drug release. The cellular uptake and antiphagocytosis ability of nanoparticles in MCF-7 tumor cells and RAW264.7 cells were investigated. The penetration ability of nanoparticles was individually evaluated in the 2D and 3D ECM models. The tissue distribution and antitumor effect of nanoparticles were evaluated in MCF-7 cell-bearing nude mice. In addition, the effects of nanoparticles on tissues and organs of mice were investigated by a hematoxylin and eosin (HE) staining method.

## Materials and methods

### Materials

Polycaprolactone-polyethylene glycol (PCL_8k_-PEG_2k_-COOH) was purchased from Shanghai Jin-pan Biological Technology Co., Ltd (Shanghai, China). Doxorubicin hydrochloride (DOX-HCl) was purchased from Shanghai Yingxuan Chempharm Co., Ltd. (Shanghai, China). Collagenase Ⅳ(Col Ⅳ) was provided by Shanghai Jinzhen Biological Technology Co., Ltd (Shanghai, China). Clusterin (CLU) was provided by Jingan Technology Co., Ltd (Shanghai, China). 3-(4,5-dimethylthiazol-2-yl)-2,5-diphenyltetrazolium bromide (MTT) was purchased from Sigma-Aldrich Co. (St. Louis, USA). 1,1'-Dioctadecyl-3,3,3',3'-tetramethylindodicarbocyanine perchlorate (DiD) was purchased from Shanghai Yi Sheng Biological Technology (Shanghai, China). Fetal bovine serum (FBS) and high-glucose Dulbecco's Modified Eagle's medium (DMEM) were purchased from Hyclone (Los Angeles, California, USA). All the cell lines used in this work were obtained from the Shanghai Institute of Biochemistry and Cell Biology (Shanghai, China). BALB/c nude mice were obtained from the Shanghai SLAC Laboratory Animal Co., Ltd. (Shanghai, China).

RAW264.7, MCF-7, and 293T cells were cultured in DMEM supplemented with 10% FBS (1% penicillin/streptomycin). Cells were incubated at 37 °C in a humidified 5% CO_2_ atmosphere. All animal experiments were performed in accordance with the institutional guidelines of the Soochow University Animal Center.

### Synthesis of PCL-PEG-ColIV

PCL-PEG-ColIV was synthesized by a carbodiimide method. Briefly, NHS (30.6 mg, 0.3 mmol) and EDC (57.4 mg, 0.3 mmol) were dissolved in 1 mL of dichloromethane and vortexed thoroughly. PCL-PEG-COOH (100 mg, 0.1 mmol) was added to 5 mL dichloromethane and stirred for 15 min until PCL-PEG-COOH was sufficiently dissolved. Then, NHS/EDC solution and PCL-PEG-COOH solution were mixed and stirred for 6 h. The intermediate (PCL-PEG-EDC) was dried under reduced pressure condition at 40 °C for 10 min. PCL-PEG-EDC suspension was obtained by dilution with 5 mL of phosphate-buffered saline (PBS) (pH 7.4) solution. PCL-PEG-ColIV was synthesized by mixing the PCL-PEG-EDC suspension and ColIV (6.5 mg) dissolved in 1 mL PBS solution (pH 7.4) and stirred for 2 h. PCL-PEG-ColIV was dialyzed in purified water for 24 h to remove the excess nonreaction materials. PCL-PEG-ColIV powder was obtained by freeze-drying at -80 °C for 48 h and stored at -20 °C.

### Preparation DOX-PCL-PEG-ColIV/CLU of nanoparticles

PCL-PEG-ColIV/CLU nanoparticles were prepared by solvent diffusion method. Briefly, 2 mg of PCL-PEG-ColIV and DOX (10:1 w/w) was dissolved in 0.5 mL acetone. To label nanoparticles, 1,1′-dioctadecyl-3,3,3′,3′-tetramethylindodicarbocyanine perchlorate (DiD) was also added to acetone at a weight ratio of 0.3% to PCL-PEG-ColIV. The above mixture was slowly added into purified water at a stirring rate of 1000 rpm, and PCL-PEG-ColIV nanoparticles were obtained by further stirring for 4 h to remove the organic solvent. Then, CLU was co-cultured with PCL-PEG-ColIV nanoparticles at the weight ratio of 1:1000 to PCL-PEG- ColIV for 1 h at 37 °C 40.

### Characterization of nanoparticles

DOX-PCL-PEG-COOH, DOX-PCL-PEG-ColIV, and DOX-PCL-PEG-ColIV/CLU nanoparticles (DOX/polymer=10:1, w/w) equivalent to 0.2 mg/mL polymer were individually prepared and dispersed in distilled water by ultrasound sonication for 30 min. The particle size and zeta potential of the samples were measured using a Zetasizer (HPP 5001, Malvern, UK). The morphology of nanoparticles was observed by transmission electron microscopy (TEM, 120 kV, HT-7700, Hitachi, Japan). The samples at 0.05 mL were deposited onto lacey carbon TEM grids and left for 2 min, dried in air at 110 °C for 5 min, and cooled to ambient temperature for morphological observation. The drug loading, and entrapment efficiency of nanoparticles were determined via dialysis method. DOX-PCL-PEG-COOH, DOX-PCL-PEG-ColIV, and DOX-PCL-PEG-ColIV/CLU nanoparticles loaded with 1 mg of DOX were dialyzed in 1000 mL of purified water for 24 h to obtain free DOX. Then, the fluorescence intensity of free DOX was measured. The encapsulation efficiency and drug loading of nanoparticles were calculated according to the following equations (1) and (2):


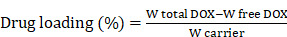
×100% (1)


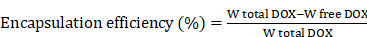
 ×100% (2)

### Drug release and cytotoxicity of nanoparticles

Two different PBS solutions (pH 7.4 and 5.5) were used as dissolution media to investigate the release characteristics of DOX, DOX-PCL-PEG-COOH, DOX-PCL-PEG-ColIV, and DOX-PCL-PEG-ColIV/CLU nanoparticles. Each sample equivalent to 1 mg DOX was placed in a dialysis bag, tied tightly with a cotton rope, suspended in a 100 mL of beaker containing 40 mL PBS, and vibrated in water-bath at 37 °C. In addition, 1 mL of dialysate was sampled at 0.5, 1, 2, 4, 8, 12, 24, 48, and 72 h, and 1 mL of blank dissolution medium preheated at 37 °C was added simultaneously. The samples were determined by a fluorescence fixed-point scanning method. The cumulative release percentage was also calculated.

The cytotoxicities of blank and drug-loaded were evaluated in different cells (293T, RAW264.7, and MCF-7). The concentration of cells was adjusted to 2×10^5^ cells/mL and seeded in 96-well culture plate for 24 h. Various concentrations of samples were obtained by diluting PCL-PEG-ColIV/CLU nanoparticles to 10, 50, 100, 500, 750, and 1000 µg/mL with DMEM. For DOX-PCL-PEG-ColIV/CLU nanoparticles, the concentrations were diluted to 5, 10, 25, 50, 100, 250, and 500 µg/mL. Then, 100 µL of each sample was added into each hole and co-cultured for 24 h (n=6). MTT solution (10 µL) at 5 mg/mL was added to each hole and co-cultured in the incubator for 4 h. The supernatant was removed, and 100 µL of dimethyl sulfoxide was added to each hole. The 96-well plate was shaken by a micro-oscillator for 10 min, and its absorbance was measured at 570 nm within 30 min by using a microplate reader. Cell viability was calculated according to the following equation (3):


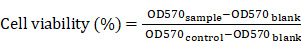
×100 (3)

### Antiphagocytosis of nanoparticles in RAW 264.7 cells

The concentration of RAW264.7 cells was adjusted to 2×10^5^ cells/mL and seeded in an observation dish for 24 h. The supernatant was removed, and RAW264.7 cells were added with 1 mL DMEM mixed with DOX, DOX-PCL-PEG-COOH or DOX-PCL-PEG-ColIV/CLU nanoparticles (n=3). The DOX concentration was 0.25 mg/mL, and the co-culture time was 2 h (37 °C, 5% CO_2_). Hoechst 33342 (10 µL/mL) of 200 µL was added to each dish and maintained for 15 min to stain the cells. Subsequently, RAW.264.7 cells were washed with PBS solution in triplicate. RAW264.7 cells were fixed with formalin (4%, v/v) for 15 min and washed with PBS solution in triplicate. Finally, all liquid in the glass dish was removed. The uptake in RAW264.7 cells was observed by confocal laser scanning microscopy (CLSM, Leica, Germany).

The concentration of RAW.264.7 cells was adjusted to 2×10^5^ cells/mL and seeded in 12-well plates for 24 h. The supernatant was removed, and 1 mL of DMEM mixed with DOX, DOX-PCL-PEG-COOH or DOX-PCL-PEG-ColIV/CLU nanoparticles was added. The DOX concentration was 0.25 mg/mL, and the co-culture time was 2 h at 37 °C. The supernatant was removed, and PBS solution was used to wash in triplicate. RAW.264.7 cells were digested by 1 mL of trypsin for 2 min in each hole and collected by centrifuging at 1000 rpm for 4 min. The supernatant was removed, and the precipitate was suspended in 0.5 mL of PBS solution. The fluorescence intensity of RAW264.7 cells was quantitatively analyzed via flow cytometry (BD FACSCalibur™, Becton Dickinson and Company, Franklin Lake, USA).

### Cellular uptake of nanoparticles in MCF-7 cells

The concentration of MCF-7 cells was adjusted to 2×10^5^ cells/mL and seeded in an observation dish for 24 h. The supernatant was removed, and MCF-7 cells were added with 1 mL of DMEM mixed with DOX, DOX-PCL-PEG-COOH or DOX-PCL-PEG-ColIV/CLU nanoparticles (n=3). The DOX concentration was 0.25 mg/mL, and the co-culture time was 2 h (37 °C, 5% CO_2_). Hoechst 33342 (10 µL/mL) of 200 µL was added to each dish for staining cell for 15 min. Subsequently, MCF-7 cells were washed with PBS solution in triplicate. MCF-7 cells were fixed with formalin (4%, v/v) for 15 min and washed again with PBS solution in triplicate. Finally, all liquid in the glass dish was removed. The uptake of MCF-7 cells was observed by CLSM.

The concentration of MCF-7 cells was adjusted to 2×10^5^ cells/mL and seeded in 12-well plates for 24 h. The supernatant was removed, and 1 mL DMEM mixed with DOX, DOX-PCL-PEG-COOH or DOX-PCL-PEG-ColIV/CLU nanoparticles (n=3) were added. The DOX concentration was 0.25 mg/mL, and the co-culture time was 2 h (37 °C, 5% CO_2_). The supernatant was removed, and sediment was washed with PBS solution in triplicate. MCF-7 cells in each hole were digested by 1 mL of trypsin for 2 min and collected by centrifuging at 1000 rpm for 4 min. The supernatant was removed, and precipitate was suspended in 0.5 mL of PBS solution. The fluorescence intensity of MCF-7 cells was quantitatively analyzed by flow cytometry.

### Penetrability of nanoparticles in 2D and 3D ECM models

The components of the 2D ECM model (Table S1) were mixed in ice bath, and the bubbles were removed by maintaining for 1 h 41. The above mixture was added to the head of quartz capillary (0.5 mm external diameter, 0.35 mm inside diameter, and 80 mm length). The head and end of quartz capillary were sealed with a sealing film and placed in water bath, which was then vibrated at 37 °C for 12 h. After the gel was formed in a quartz capillary tube, the 2D ECM model was established and used for penetration study.

Ten microliters of DOX-PCL-PEG-COOH, DOX-PCL-PEG-ColIV, and DOX-PCL-PEG-ColIV/CLU nanoparticles (equivalent to 1 mg/mL of DOX) were injected into the head of quartz capillary and co-cultured for 3 h. The fluorescence intensity of the head, middle, and end of quartz capillary was observed via CLSM 42.

The components of the 3D ECM model, chitosan solution (0.24% w/w), and gelatin coating solution (0.24% w/w) were mixed at the ratio of 1 to 3, and the pH was adjusted to neutral using 1 N NaOH solution in an ice bath (Table S2). The resulting mixture (500 µL) was added to each well in 12 well plates (n=3). After 2 h of ultraviolet sterilization, the mixture was placed in 5% CO_2_ cell incubator at 37 °C and maintained for 30 min to form the gel matrix. Meanwhile, MCF-7 cells were digested with a count of 2×10^5^ cells/mL. Then, 2 mL of the cellular suspension was incubated with the gel matrix in each well for 24 h. The 3D ECM model was observed and photographed by an optical microscope 43,44.

One hundred microliters of DOX-PCL-PEG-COOH, DOX-PCL-PEG-ColIV, and DOX-PCL-ColIV/CLU nanoparticles (1 mg/mL of the polymer) was added into each well and co-cultured in a 5% CO_2_ incubator at 37 °C for 2 h. Hoechst33342 (10 µL/mL) at 200 µL was used to stain the MCF-7 cells for 15 min. Then, the suspension of MCF-7 cells was collected and centrifuged at 200 rpm for 5 min. The supernatant was removed, and the precipitate was suspended with 200 µL of PBS again. The appropriate amount of cellular suspension was dropped on the glass slide and dried for 1 h in the fume cupboard. The dried MCF-7 cells were covered with a clean cover glass. The uptake of MCF-7 cells in the 3D ECM model was observed using CLSM.

### *In vivo* fluorescent imaging

The mouse breast tumor model was established by subcutaneous inoculating 200 µL of MCF-7 cells (5×10^7^ cells/mL) into the right armpit of female BALB/c nude mice at 2-4 weeks old. After 2 weeks, tumor volume reached 50-80 mm^3^. MCF-7 cell-bearing nude mice were randomly divided into four groups (n=3). DiD-labeled DOX-PCL-PEG-COOH, DOX-PCL-PEG-ColIV, and DOX-PCL-PEG-ColIV/CLU (10 mg/mL) nanoparticles or saline of 150 µL was injected through the tail vein, and the DiD fluorescence was observed at predetermined time points (0, 12, 24, 48, and 72 h) by a small animal imaging system (Caliper IVIS Lumina II, Xenogen, USA). The excitation wavelength was 640 nm, and the emission wavelength was 680 nm. After 72 h, MCF-7 cell-bearing nude mice were sacrificed by cervical dislocation, and the main organs including the heart, liver, spleen, lung, kidney, and tumor tissues were obtained. The tissues were washed with PBS. Then, the bio-distributions of DiD-labeled nanoparticles were observed and calculated using IVIS imaging analysis software.

### *In vivo* antitumor effect

MCF-7 cell-bearing nude mice were randomly divided into four groups (n=6). DOX-PCL-PEG-COOH, DOX-PCL-PEG-ColIV, and DOX-PCL-PEG-ColIV/CLU nanoparticles or saline were injected via tail vein. The DOX dose of each group was 5 mg/kg. The mice were administered every 2 days for 10 days. The changes of mouse body weight and tumor size were recorded throughout the whole experiment.

### Histological study

After treatment, MCF-7 cell-bearing nude mice were sacrificed by cervical dislocation. The main tissues and organs, including the heart, liver, spleen, lung, kidney, and tumor were obtained, cleaned with PBS solution, and fixed with 10% formalin solution, which were then embedded in paraffin and cut into slices. The slices were H&E stained. Subsequently, the changes of tissues and organs were observed using an ordinary optical microscope.

In addition, the expression of collagen IV in tumor specimens was also analyzed. The tumor specimens isolated from saline and DOX-PCL-PEG-ColIV/CLU nanoparticle groups were fixed in 4% paraformaldehyde and incubated with anti-IV collagen antibody.

### Statistical analysis

All data were presented as means±standard deviation. The statistical analysis of the samples was performed via Student's t-test by using SPSS Statistics 17.0 software. A p-value below 0.05 was considered statistically significant.

## Results and Discussion

### Preparation of DOX-PCL-PEG-ColIV/CLU nanoparticles

PCL-PEG-ColIV was synthesized by linking PCL-PEG-COOH and ColIV (160 U/mg) via a carbodiimide method (Figure S1A). The brown powder of PCL-PEG-ColIV was formed and stored at -20 °C (Figure S1B). The significant change of melting point measured by differential scanning calorimetry (DSC) meant that ColIV-modified PCL-PEG (PCL-PEG-ColIV) had been successfully synthesized (Figure S2). The critical micelle concentrations (CMCs) of PCL-PEG-COOH and PCL-PEG-ColIV indicated the PCL-PEG-ColIV formation (Figure S3).

For detecting the combination rate of ColIV, the calibration curve of ColIV was drawn (Figure S4A), and the calculated combination rate of ColIV was 96%. The unconjugated ColIV was removed via dialysis. Enzyme ColIV activity in nanoparticles was determined by ninhydrin colorimetry and the calibration curve of glycine was drawn (Figure S4B). The ColIV activity reached up to 99.74%. DOX-loaded nanoparticles (DOX-PCL-PEG-ColIV) were prepared by self-assembly, and the calibration curve of DOX was drawn at the concentration range of 1-5 µg/mL for the detection of drug loading and encapsulation efficiency (Figure S4C). DOX-PCL-PEG-ColIV/CLU nanoparticles were prepared by adsorbing CLU on the surface of DOX-PCL-PEG-ColIV nanoparticles. Bovine serum albumin (BSA) was commonly used as a standard substance in the determination of CLU concentration. The calibration curve of BSA showed a good linearity in the concentration range of 0-50 µg/mL, and the binding rate of CLU on DOX-PCL-PEG-ColIV nanoparticles was 84.54% (Figure S4D).

### Characterization of DOX-PCL-PEG-ColIV/CLU nanoparticles

The appearance, TEM images, particle size, and zeta potential of three blank nanoparticle solutions (PCL-PEG-COOH, PCL-PEG-ColIV, and PCL-PEG-ColIV/CLU nanoparticles) were preliminarily investigated. All three blank nanoparticle solutions were clear and transparent (Figure S5A), and they had regular spherical structure (Figure S5B). The particle size of PCL-PEG-ColIV nanoparticles increased from 68.0 nm to 150.4 nm after modification with ColIV. Such increase was related to the increase of molecular weight of enzymes. After further adsorption with CLU, the particle size was 151.8 nm. The zeta potentials of all three nanoparticles were approximately -20 mV (Figure S5C).

**Figure 1:**
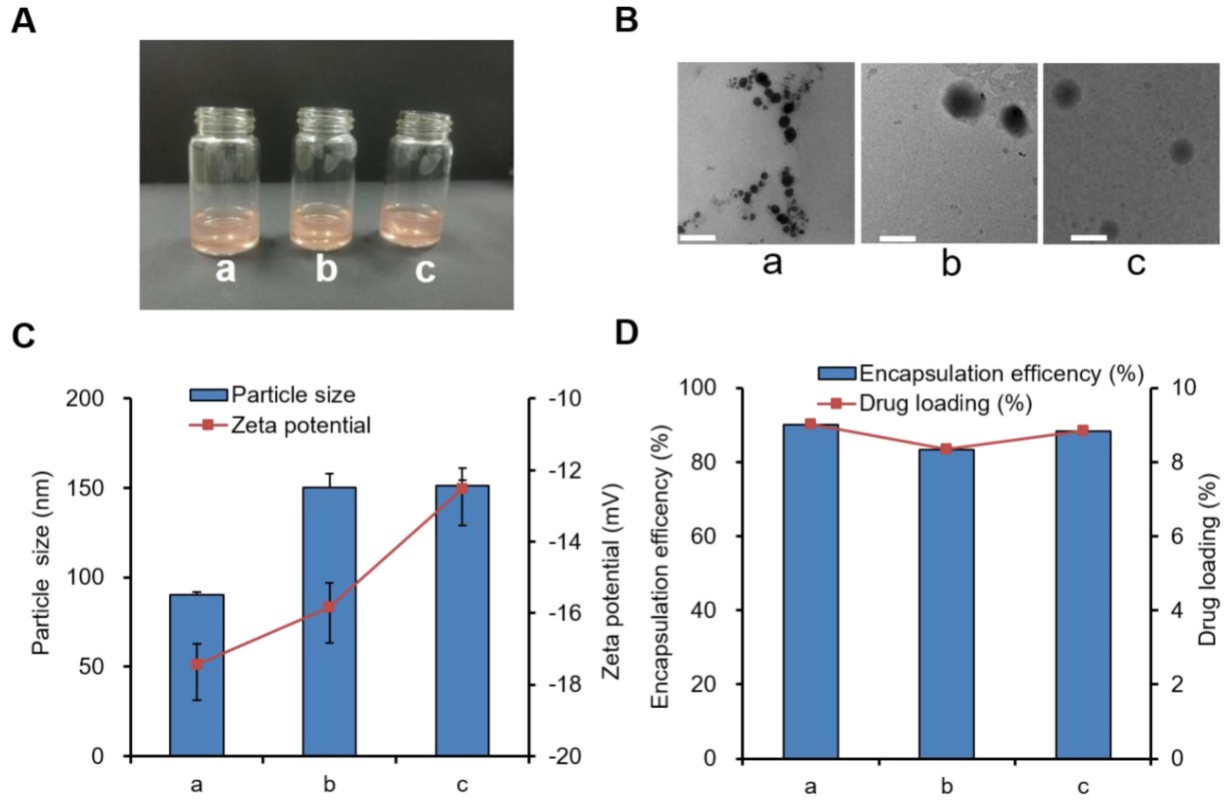
Three types of drug-loaded nanoparticle solutions (a) DOX-PCL-PEG-COOH; (b) DOX-PCL-PEG-ColIV; (c) DOX-PCL-PEG-ColIV/CLU). (A) Appearance. (B) TEM images. The scale bar represents 100 nm. (C) Particle size and zeta potential. (D) Drug loading capacity and encapsulation efficiency.

We further explored the characterization of three drug-loading nanoparticles, including DOX-PCL-PEG-COOH, DOX-PCL-PEG-ColIV, and DOX-PCL-PEG-ColIV/CLU nanoparticles. The appearance of all three drug-loading nanoparticle solutions was also clear and red transparent, as shown in Figure 1A, and they were similar to blank nanoparticles in Figure 1B. Fourier-transform infrared spectroscopy (FT-IR) results proved that DOX had been encapsulated into PCL-PEG-COOH and PCL-PEG-ColIV nanoparticles (Figure S6). After modification of ColIV, the particle size of DOX-PCL-PEG-ColIV nanoparticles increased from 90.1 nm to 150.2 nm, and such an increase was related to the fact that PCL-PEG modification facilitates the formation of larger aggregates/supramolecular assemblies. After further CLU adsorption, the particle size of DOX-PCL-PEG-ColIV/CLU was 151.4 nm without a significant difference compared with DOX-PCL-PEG-ColIV. All three nanoparticles had negative charge zeta potentials in the range of -12--18 mV, and their absolute values had minimal difference (Figure 1C). The particle sizes and zeta potentials in drug-loaded nanoparticles were close to those of three blank nanoparticles. As shown in Figure 1D, the DOX loading capacity of PCL-PEG-COOH nanoparticles was 9.01%, similar to DOX-PCL-PEG-ColIV nanoparticles (8.34%) and PCL-PEG-ColIV/CLU nanoparticles (8.84%). Meanwhile, the encapsulation efficacies were 90.09% for DOX-PCL-PEG-COOH nanoparticles, 83.38% for DOX-PCL-PEG-ColIV nanoparticles, and 88.36% for DOX-PCL-PEG-ColIV/CLU nanoparticles, thereby showing a relatively high encapsulation efficiency. No significant difference was observed in the drug loading between each group (p>0.05).

### *In vitro* drug release and cytotoxicity of DOX-PCL-PEG-ColIV/CLU nanoparticles

**Figure 2:**
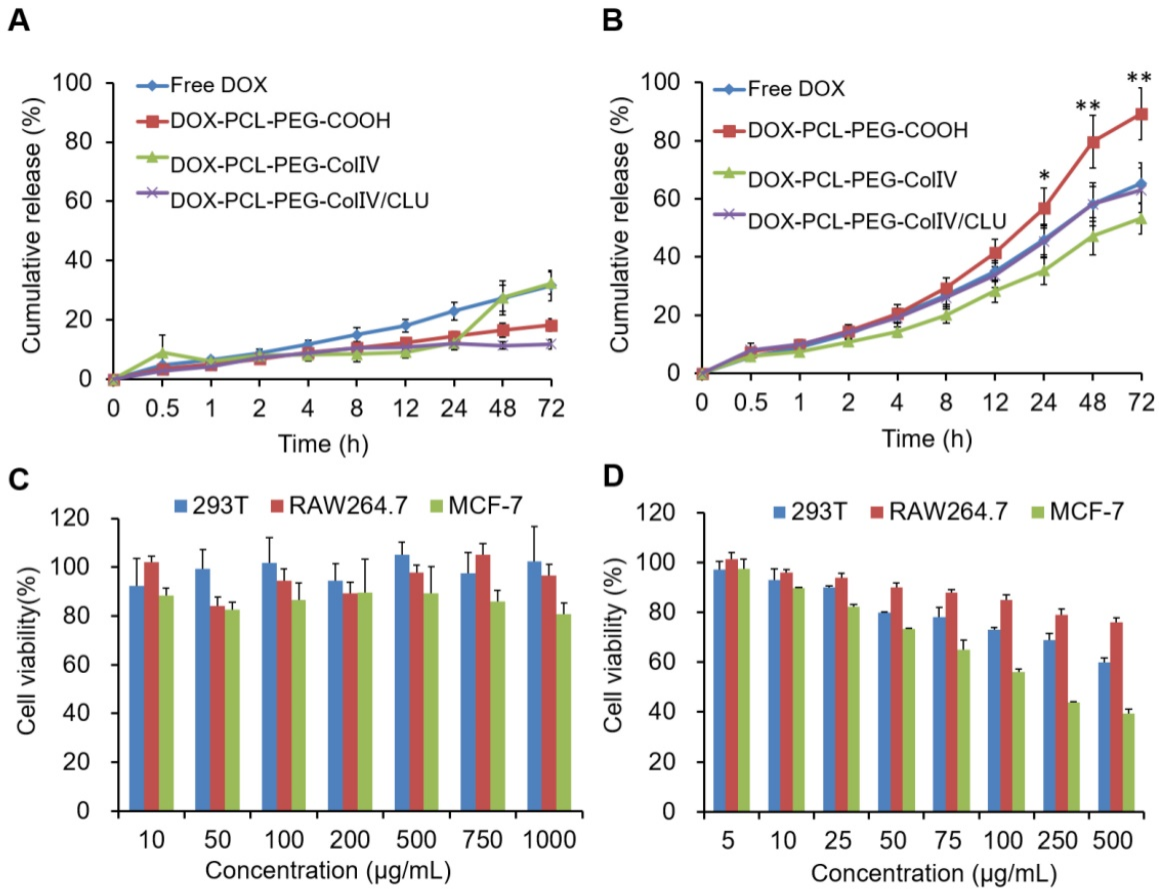
(A) *In vitro* drug release in PBS buffer solutions at pH 7.4. (B) *In vitro* drug release in PBS buffer solutions at pH 5.5. (C) Cell viability of different cell types co-cultured with PCL-PEG-ColIV/CLU nanoparticles for 24 h via the MTT method. 293T cells (blue bar), RAW264.7 cells (red bar), and MCF-7 cells (green bar). (D) Cytotoxicity of DOX-PCL-PEG-ColIV/CLU nanoparticles in 293T, RAW264.7, and MCF-7 cells. ^*^p < 0.05, ^**^p < 0.01 compared with free DOX group.

In order to compare the drug release from nanoparticles in the normal physiological fluid (pH7.4) and tumor tissues (pH5.5), two PBS solutions with different pH levels (pH7.4 and 5.5) were used as dissolution media. The release study was carried out by a dialysis bag method. The release rate of DOX (pure drug), DOX-PCL-PEG-COOH, DOX-PCL-PEG-ColIV, and DOX-PCL-PEG-ColIV/CLU nanoparticles in PBS solutions are shown in Figure 2A and 2B. The pure drug presented a remarkable higher drug release at pH5.5 (32%) than at pH7.4 (65%) at 72h due to its pH dependent solubility. DOX-PCL-PEG-COOH nanoparticles, DOX-PCL-PEG-ColIV nanoparticles, and DOX-PCL-PEG-ColIV/CLU nanoparticles were similar to DOX in pH 7.4 and 5.5 media. At pH 7.4, the release medium cannot form a sink condition, so no different release profiles were observed between the drug and drug-loaded nanoparticles. However, at pH5.5, a relatively high drug release was found for DOX-PCL-PEG-COOH nanoparticles compared with other groups. This may be attributed to the fact that DOX is highly dispersed in the PCL layer at a molecular level, thereby exhibiting higher drug release than free DOX. The release of DOX depends on the pH value of the release media. The tumor cells are in slightly acidic microenvironment, conducive to the release of DOX from nanoparticles in acidic tumor site 45.

The safety of blank PCL-PEG-ColIV/CLU nanoparticles was investigated via MTT method for three different cell types, namely, 293T, RAW264.7, and MCF-7 cells. As shown in Figure 2C, the cellular survival rates of PCL-PEG-ColIV/CLU nanoparticles co-cultured with 293T cells, RAW264.7 cells, and MCF-7 cells after 24 h were higher than 80% in the concentration range of 10-1000 µg/mL, thereby showing satisfactory biological safety. The cytotoxicity of DOX-PCL-PEG-ColIV/CLU nanoparticles to 293T cells, RAW264.7 cells, and MCF-7 cells was further explored in the concentration gradients of 5, 10, 25, 50, 100, 250, and 500 µg/mL in Figure 2D. The IC_50_ values of DOX-PCL-PEG-ColIV/CLU nanoparticles to 293T cells, RAW264.7 cells, and MCF-7 cells were 524.29 µg/mL, 795.38 µg/mL, and 147.89 µg/mL, respectively. The drug-loaded nanoparticles significantly promote the apoptosis of MCF-7 tumor cells, showing relatively low toxicity in 293T cells and RAW264.7 cells.

### Antiphagocytosis of DOX-PCL-PEG-ColIV/CLU nanoparticles

**Figure 3:**
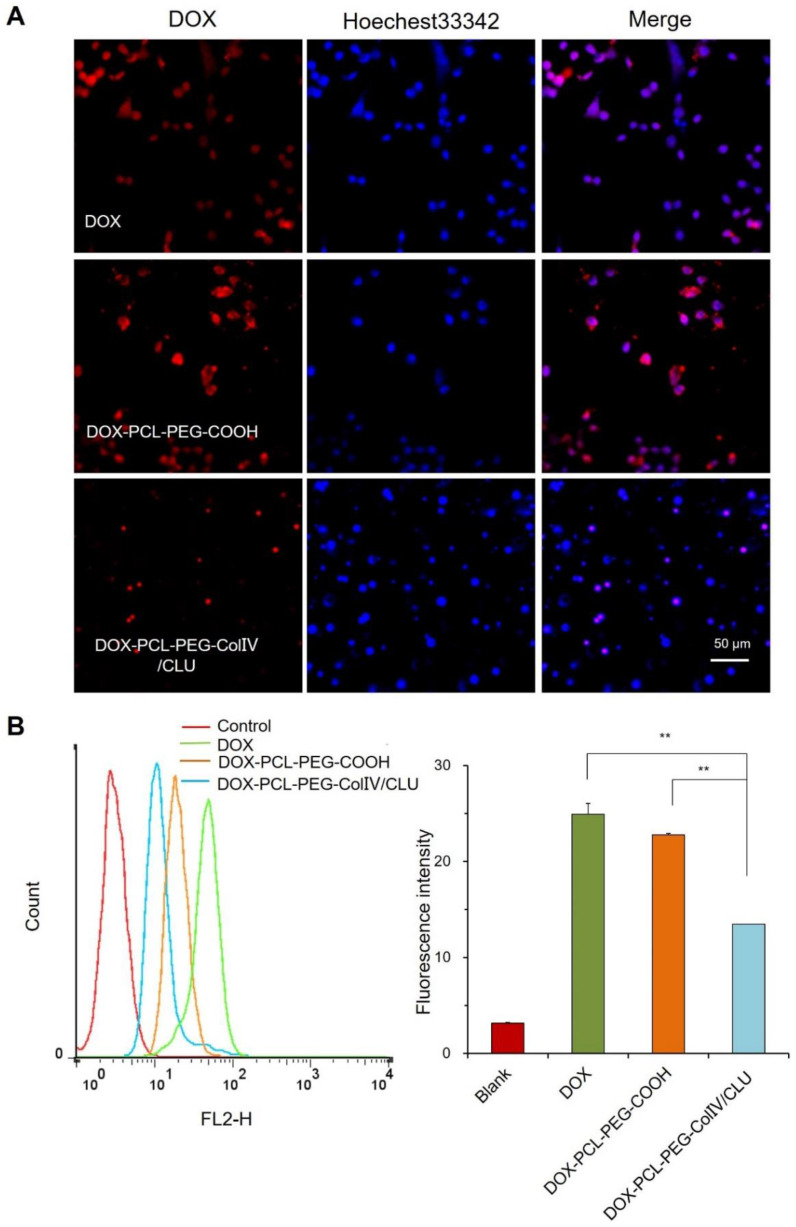
Cellular uptake of different carriers in RAW 264.7 cells after incubation for 2 h. (A) Confocal laser scanning microscopy. (B) Flow cytometry (n=3). The cellular uptakes of free DOX, DOX-PCL-PEG-COOH, and DOX-PCL-PEG-ColIV/CLU nanoparticles were compared (^**^p < 0.01).

To explore whether the DOX-PCL-PEG-ColIV/CLU nanoparticles had antiphagocytosis ability, the cellular uptake of DOX, DOX-PCL-PEG-COOH, and DOX-PCL-PEG-ColIV/CLU nanoparticles in RAW264.7 cells was determined using CLSM. As shown in Figure 3A, DOX and DOX-PCL-PEG-COOH nanoparticles were both easily observed in RAW264.7. By contrast, DOX-PCL-PEG-ColIV/CLU nanoparticles significantly reduced the cellular uptake. These results indicated that DOX-PCL-PEG-ColIV/CLU nanoparticles could reduce the phagocytosis of nanoparticles from RAW264.7 cells.

The cellular uptake of DOX, DOX-PCL-PEG-COOH, and DOX-PCL-PEG-ColIV/CLU nanoparticles in RAW264.7 cells was also determined via flow cytometry. The average fluorescence intensity of DOX and DOX-PCL-PEG-COOH nanoparticles both increased significantly (p < 0.01) compared with the DOX-PCL-PEG-ColIV/CLU nanoparticles (Figure 3B). These results evidently showed that DOX-PCL-PEG-ColIV/CLU nanoparticles had an antiphagocytosis effect on RAW264.7 cells. This condition might decrease the specific uptake of nanocarriers by the main organs *in vivo*. Clusterin has been known as the major protein in the corona of both polymer-modified nanocarriers, with its highest enrichment on PEGylated surfaces. Furthermore, pre-loading of the PEGylated nanocarriers with clusterin reduced macrophage uptake, providing evidence for a dysopsonizing function of clusterin concerning internalization into macrophages 34.

### Cellular uptake of DOX-PCL-PEG-ColIV/CLU nanoparticles

**Figure 4:**
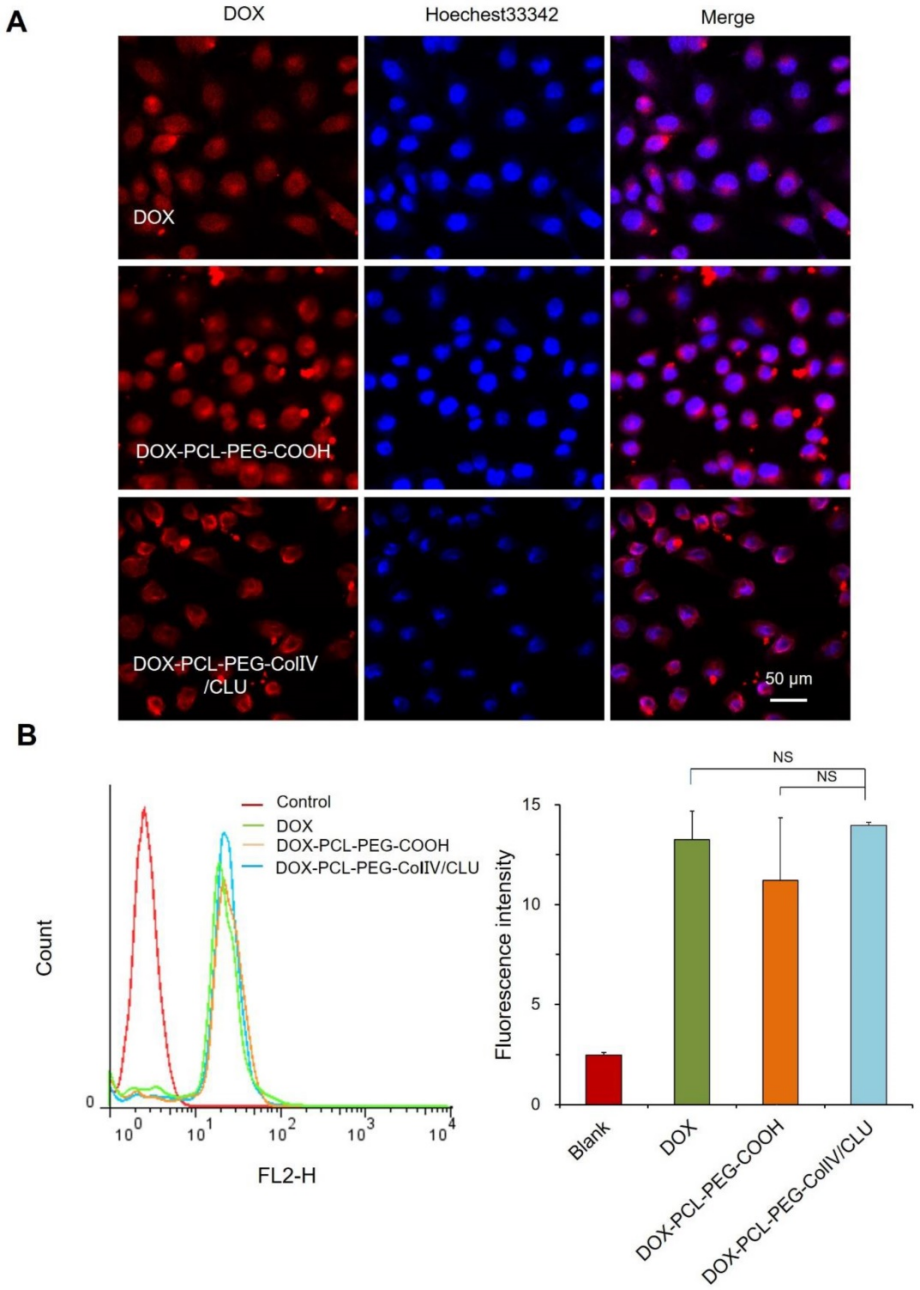
Cellular uptake of different carriers in MCF-7 cells after incubation for 2h. (A) Confocal laser scanning microscopy. (B) Flow cytometry. The cellular uptakes of DOX, DOX-PCL-PEG-COOH, and DOX-PCL-PEG-ColIV/CLU nanoparticles were observed using CLSM after co-cultivation with MCF-7 cells for 2 h (^NS^ no significant difference).

To investigate the internalization of nanoparticles with the MCF-7 cells, the cellular uptakes of DOX, DOX-PCL-PEG-COOH, and DOX-PCL-PEG-ColIV/CLU nanoparticles were observed using CLSM after co-cultivation with MCF-7 cells for 2 h. DOX, DOX-PCL-PEG-COOH, and DOX-PCL-PEG-ColIV/CLU nanoparticles were almost completely taken up by the MCF-7 cells (Figure 4A). The ColIV and CLU-modified nanoparticles did not affect the uptake of MCF-7 cells.

The internalization of DOX, DOX-PCL-PEG-COOH, and DOX-PCL-PEG-ColIV/CLU nanoparticles in MCF-7 cells was further quantitatively detected via flow cytometry. The average fluorescence intensity of the three groups ranged from 12 to 15, and no significant difference was observed (Figure 4B). ColIV and CLU-modified nanoparticles did not affect the uptake of nanocarriers by tumor cells. The relative fluorescent intensity of DOX-PCL-PEG-COOH in RAW 264.7 cells (Figure 3) was higher than that in MCF7 cells (Figure 4) because the unmodified nanoparticles have higher abilities of macrophage (RAW 264.7 cells) uptake than the tumor cell uptake.

### Penetrability of nanoparticles in the 2D and 3D ECM models

Gels are composed of HA (5 mg/mL). The collagen type IV was derived from rat tail (5 mg/mL), and gelatin (2.4 mg/mL) served as the 2D ECM model. The white translucent gels could also be formed (Figure S7A). The capillary was filled with gels, wherein the ColIV effect on penetrability was detected. The fluorescence of three parts of capillary was observed using CLSM, including the head, middle, and terminal parts.

**Figure 5:**
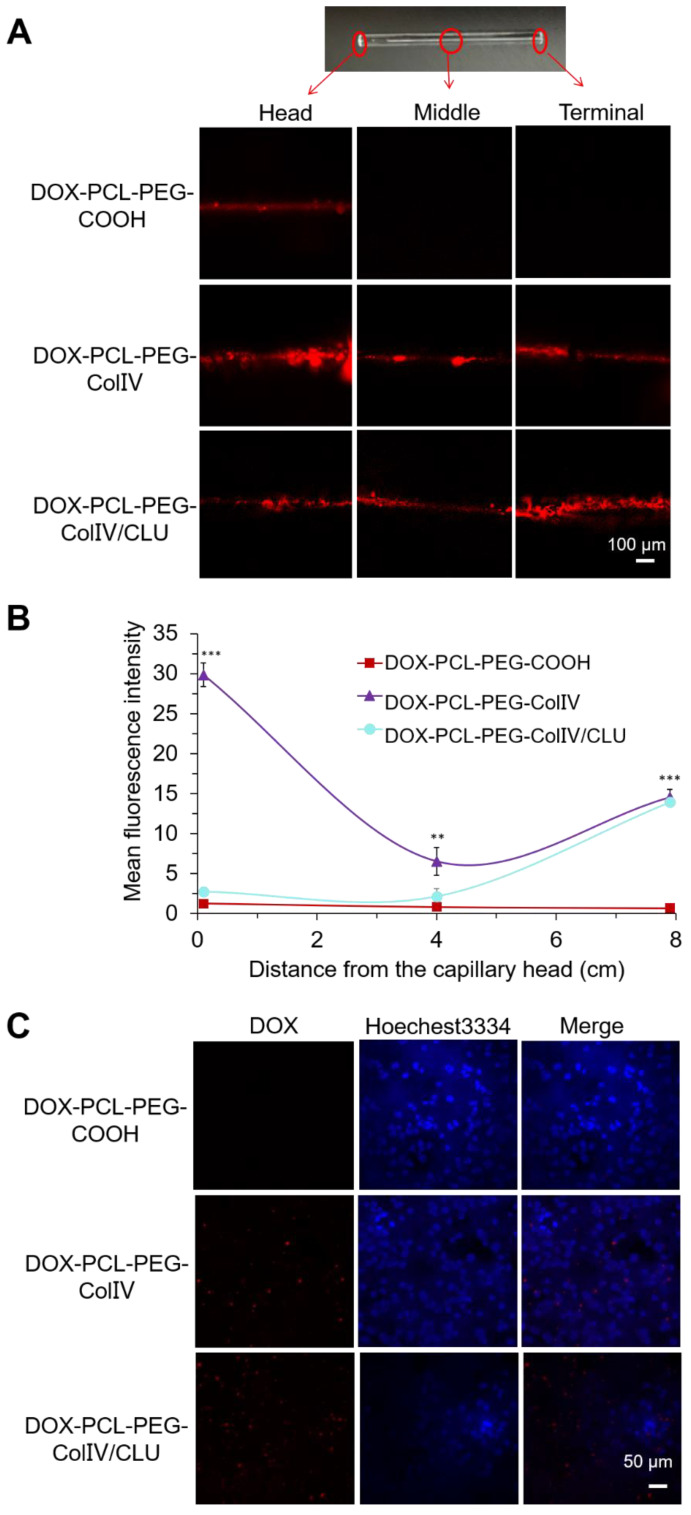
Penetration ability of various drug-loaded nanocarriers in the 2D and 3D ECM models. (A) Confocal images in the 2D ECM model (ColIV= 10 U/mL). (B) Mean fluorescence intensity *vs.* distance from the capillary head. ^**^p < 0.01, ^***^p < 0.001. (C) Confocal images in the 3D ECM model (ColIV= 10 U/mL).

The penetration of DOX-PCL-PEG-COOH, DOX-PCL-PEG-ColIV, and DOX-PCL-PEG-ColIV/CLU nanoparticles in the 2D ECM model is shown in Figure 5A. In the DOX-PCL-PEG-COOH nanoparticle group, DOX fluorescence only appeared in the middle, whereas DOX fluorescence penetrated through the whole capillary in the nanoparticles modified with ColIV (10 U). As shown in Figure 5B, both DOX-PCL-PEG-ColIV and DOX-PCL-PEG-ColIV/CLU nanoparticles showed significantly higher fluorescence intensity than DOX-PCL-PEG-COOH at the terminal site of the capillary(p < 0.001), while only DOX-PCL-PEG-ColIV had a remarkable increase of fluorescence signal at the head and middle sites of the capillary (p < 0.001, p < 0.01). Even interesting, DOX-PCL-PEG-COOH nanoparticles physically mixed with the amount of effective ColIV (10 U or 100 U) simply penetrate into the middle of the 2D ECM gel in the capillary, but the nanoparticles physically mixed with 1000 U of ColIV penetrated through the three parts of the capillary (Figure S8). The nanocarrier-conjugated ColIV can significantly increase the penetration for ECM gels. These data showed that the nanoparticles chemically modified with ColIV had stronger penetration ability than the nanoparticles physically mixed with the same amount of ColIV.

To further explore the effect of ColIV on penetrability, the 3D ECM model was also established. Most MCF-7 cells in the ECM 3D model were suspended and clustered (Figure S7B), whereas the MCF-7 cells cultured on 2D plates were adhere-wall. The significant differences in morphology between the 2D and 3D ECM models and the 3D ECM model well simulated the actual living environment of tumor cells *in vivo*.

The penetration of DOX-PCL-PEG-COOH, DOX-PCL-PEG-ColIV, and DOX-PCL-PEG-ColIV/CLU nanoparticles in the 3D ECM model was imaged by CLSM in Figure 5C. DOX fluorescence was not obvious in the DOX-PCL-PEG-COOH nanoparticle group, but DOX fluorescence significantly increased in the groups of DOX-PCL-PEG-ColIV and DOX-PCL-PEG-ColIV/CLU nanoparticles. The ColIV effectively increased the penetration of nanoparticles to tumor ECM and delivered several DOX to the tumor site.

### *In vivo* fluorescent imaging

**Figure 6:**
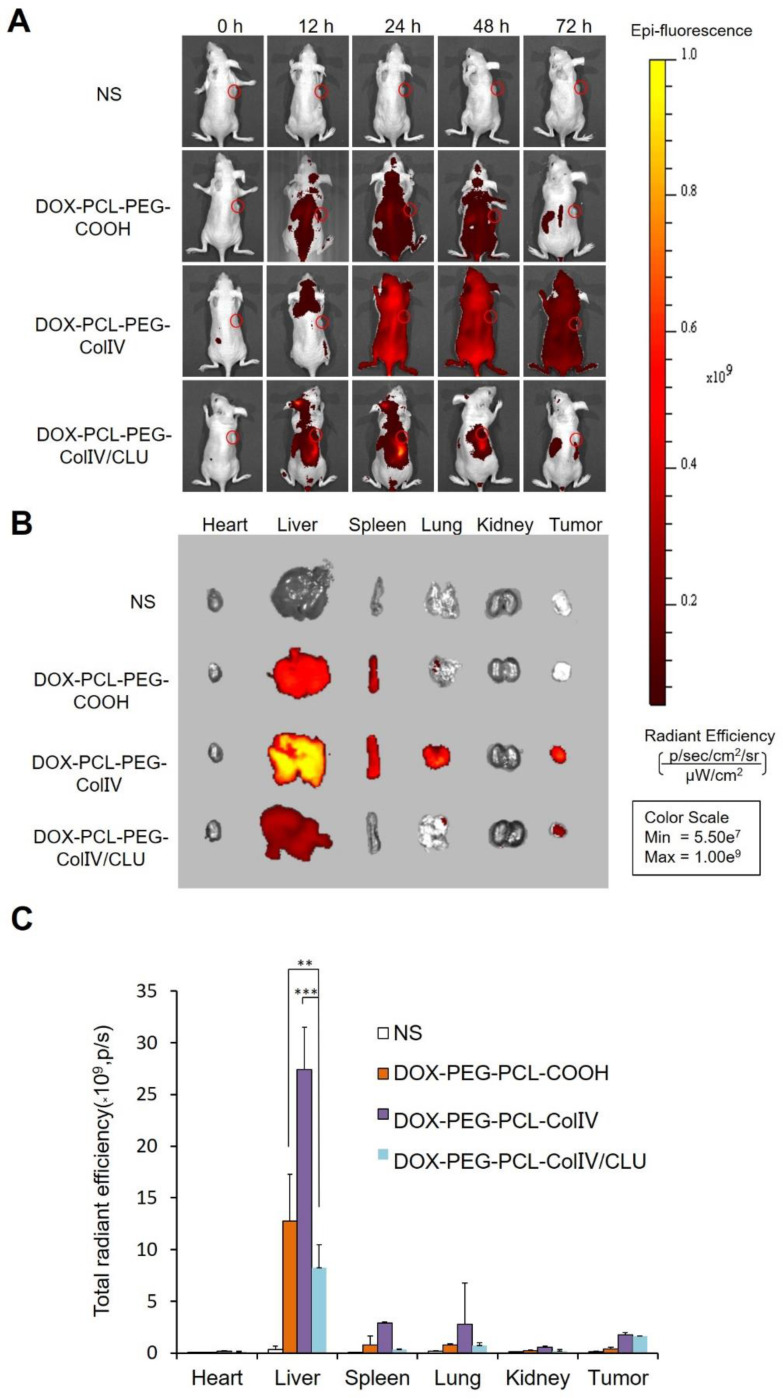
Tissue distribution. (A) *In vivo* fluorescence images of MCF-7 tumor-bearing nude mice after administration of nanoparticles through the tail vein at 0, 12, 24, 48, and 72 h. (B) *In vivo* fluorescence images of various organs after administration of nanoparticles through the tail vein at 72 h. (C) Radiant efficiency of fluorescence signals, **p < 0.01, ***p < 0.001.

MCF-7 cell-bearing nude mice, which were used to study the biodistribution of DiD-labeled nanoparticles, were employed to mimic human breast cancer. The DiD signals of NS, DOX-PCL-PEG-COOH, DOX-PCL-PEG-ColIV, and DOX-PCL-PEG-ColIV/CLU nanoparticles were detected after injection of 12, 24, 48, and 72 h by using a small animal imaging system. As shown in Figure 6A, DOX-PCL-PEG-COOH and DOX-PCL-PEG-ColIV nanoparticles were distributed throughout the body at 24 and 48 h, and the fluorescence of DOX-PCL-PEG-COOH nanoparticles almost completely disappeared, but DOX-PCL-PEG-ColIV nanoparticles still had high fluorescence at 72 h. However, the fluorescence intensity of DOX-PCL-PEG-ColIV nanoparticles was selective and higher in the liver and tumor sites than the other two groups over time. The DOX-PCL-PEG-ColIV nanoparticles could penetrate all collagen-rich tissues and organs along with the blood circulation. Although the liver has a very low concentration of collage, the nanoparticle accumulated highly in the liver. This might be due to the high blood stream flow in the liver compared to other organs, which can perfuse and motivate the transport and uptake of DOX-PCL-PEG-ColIV nanoparticles. By contrast, the distribution of DOX-PCL-PEG-ColIV/CLU nanoparticles had a distinct selectivity. The fluorescence intensity of DOX-PCL-PEG-ColIV/CLU nanoparticles mainly concentrated in the liver and tumor, whereas fluorescent signals were seldom found in the spleen and lung because of the protein corona formed on the surface of nanoparticles. Although nanoparticles modified with PEG with stealth effect can reduce the binding of proteins on the surface of nanoparticles and help avoid the rapid recognition by the reticular endothelial system, nanoparticles may still be recognized. Thus, complete avoidance of liver uptake was rarely possible 46,47.

At 72 h post of tail vein injection of nanoparticles, the mice were sacrificed, and the distribution in the main organs and tumor tissue was analyzed in Figure 6B. Their radiant efficiency was also quantified in Figure 6C. In the liver, the fluorescence intensity of the DOX-PCL-PEG-ColIV nanoparticle group (p < 0.01) and the DOX-PCL-PEG-COOH nanoparticle group (p < 0.001) significantly increased compared with DOX-PCL-PEG-ColIV/CLU nanoparticles. This result further illustrated that DOX-PCL-PEG-ColIV nanoparticles were taken up rapidly in the liver. By contrast, DOX-PCL-PEG-ColIV/CLU nanoparticles were taken up relatively slowly and had antiphagocytosis effect. In the tumor tissue, the DOX-PCL-PEG-ColIV nanoparticle and DOX-PCL-PEG-ColIV/CLU nanoparticle groups had more fluorescence than the DOX-PCL-PEG-COOH nanoparticle group. The results were consistent with Figure 6A, thereby further showing that DOX-PCL-PEG-ColIV/CLU nanoparticles had antiphagocytosis effect and helped deliver the drugs into the tumor.

### *In vivo* antitumor efficacy

**Figure 7:**
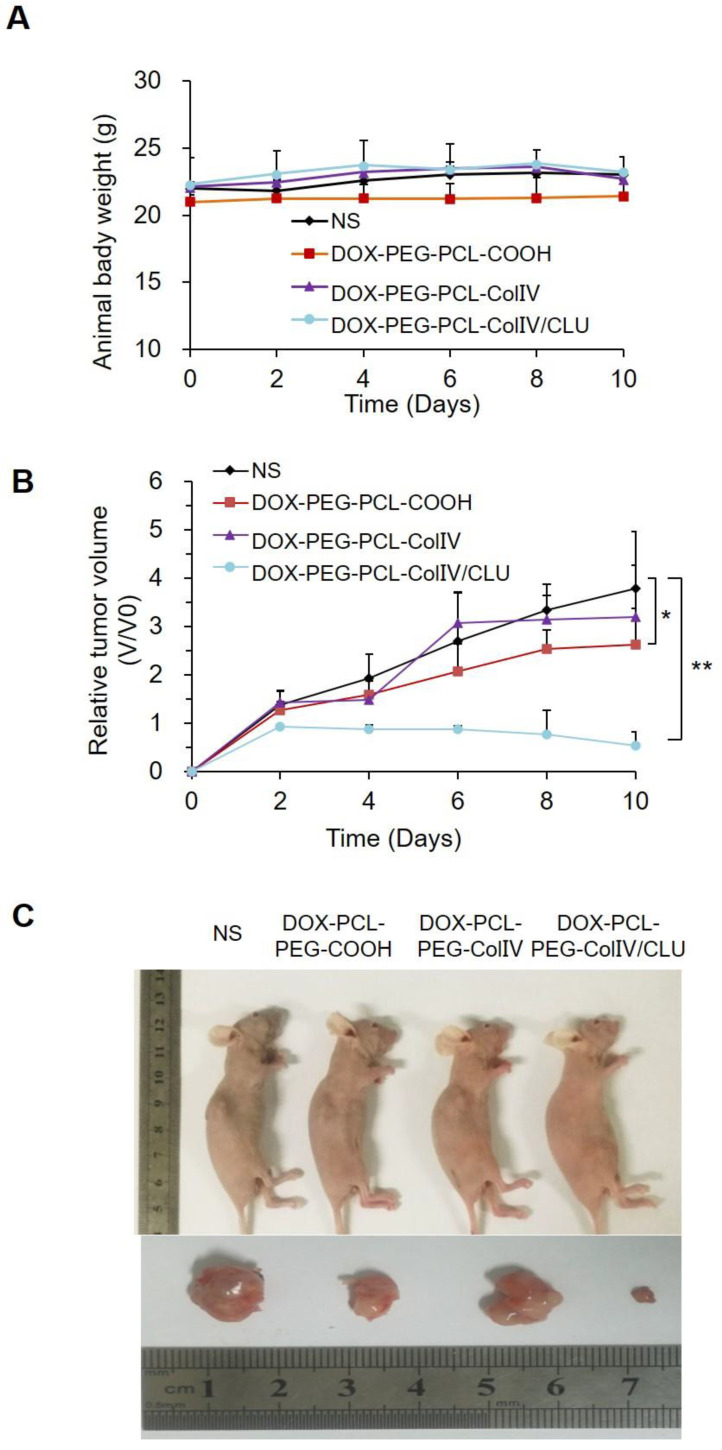
*In vivo* antitumor efficacy. (A) Changes of body weight in tumor-bearing nude mice. (B) Changes of body weight in tumor-bearing nude mice, *p < 0.05, **p < 0.01. (C) Comparison of tumor volume in tumor-bearing nude mice.

MCF-7 cell-bearing nude mice were randomly divided into four groups, namely, the NS, DOX-PCL-PEG-COOH, DOX-PCL-PEG-ColIV, and DOX-PCL-PEG-ColIV/CLU nanoparticle groups. The body reactions and body weight changes were monitored after tail vein injection every 2 days for 10 consecutive days. The mice in the four groups had no abnormalities, such as high frequency tremor after 2 h of administration. The body weight of NS, DOX-PCL-PEG-COOH, DOX-PCL-PEG-ColIV, and DOX-PCL-PEG-Col IV/CLU nanoparticles were 22.01±0.44, 20.99±0.55, 22.14±0.35, and 22.32±2.01 g at 0 days, and 23.07±0.07, 21.44±1.16, 22.69±0.84, and 23.09±1.01 g at 10 days, respectively (Figure 7A). The body weight of mice in all groups did not significantly change; thereby suggesting that the toxicity of DOX loaded nanoparticles was relatively small to nude mice.

The change of tumor volume in the four groups was recorded every 2 days for 10 consecutive days, as shown in Figure 7B. The tumor tissues were removed from the body for imaging at 10 days in Figure 7C. Compared with the NS group, the tumor volume of the DOX-PCL-PEG-COOH nanoparticle group was significantly different (p < 0.05), but the tumor volume of DOX-PCL-PEG-ColIV nanoparticle group did not show any difference (p>0.05). By contrast, the tumor volume of the DOX-PCL-PEG-ColIV/CLU nanoparticle group remarkably decreased and showed a significant difference (p < 0.01). In addition, the tumor volume significantly decreased for DOX-PCL-PEG-ColIV/CLU nanoparticles, whereas those of DOX-PCL-PEG-COOH and DOX-PCL-PEG-ColIV nanoparticles increased.

However, the changes of tumor size in the DOX-PCL-PEG-COOH and DOX-PCL-PEG-ColIV nanoparticle groups were not consistent with the results of fluorescence distribution in nude mice after 72 h of tail vein administration. Therefore, this study further investigated the tissue fluorescence distribution of nude mice after 12 h of administration. As shown in Figure S9, DOX-PCL-PEG-COOH nanoparticles had obvious fluorescence, but DOX-PCL-PEG-ColIV nanoparticles had less fluorescence in the tumor tissue. This condition speculated that DOX-PCL-PEG-COOH nanoparticles experienced less phagocytosis caused by the PEG chain on the surface at 12 h, thereby resulting in an increased uptake at the tumor site. However, due to the good penetration of ColIV, DOX-PCL-PEG-ColIV nanoparticles penetrate the visceral tissues rich in collagen at 12 h, thereby resulting in a remarkable decrease in the uptake of DOX-PCL-PEG-ColIV nanoparticles at the tumor site. Meanwhile, the fluorescence intensity of DOX-PCL-PEG-COOH nanoparticles decreased with the growth of tumors at 72 h, whereas DOX-PCL-PEG-ColIV nanoparticles redistributed from other organs to tumors, thereby resulting in significant accumulation of tumors. The main reason why the antitumor effect of DOX-PCL-PEG-ColIV nanoparticles was lower than that of DOX-PCL-PEG-COOH nanoparticle was that the dynamic equilibrium of distribution in different kinds of nanoparticles differed *in vivo*.

### Histological study

**Figure 8:**
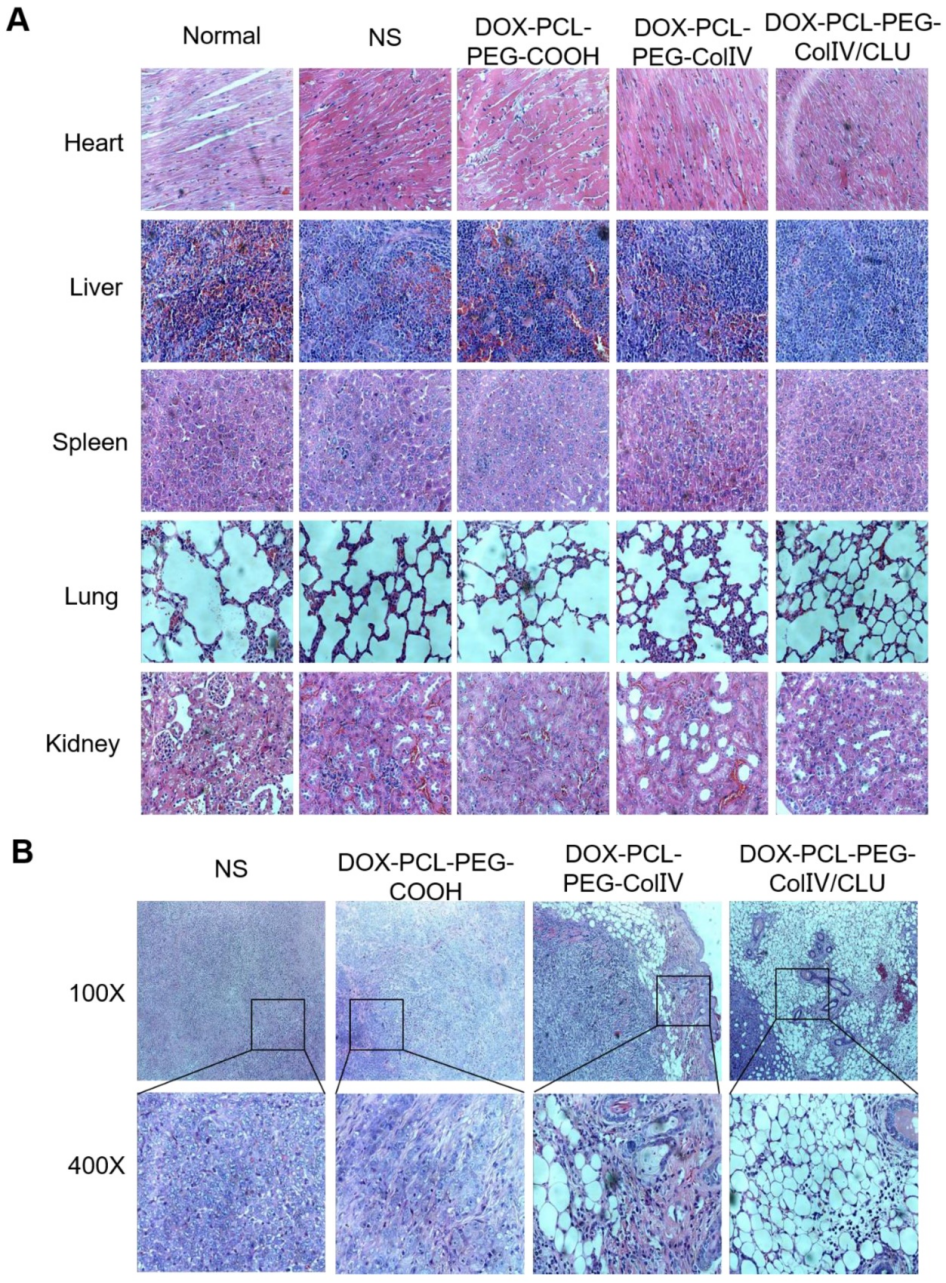
Histological HE staining assessments of major organs and tumor tissues in tumor-bearing nude mice. (A) Major organs (400×). (B) Tumor site (100× and 400×). Black boxes in the 100× group were scaled-down versions of the images in the 400× group.

The major tissues of MCF-7 cell-bearing nude mice after the treatment were stained for histological observation, as shown in Figure 8A. Compared with the normal group, the DOX-PCL-PEG-COOH, DOX-PCL-PEG-ColIV, and DOX-PCL-PEG-ColIV/CLU nanoparticle groups showed no obvious histological changes in the main organs, including the heart, liver, spleen, lung, and kidney, during the treatment. Although DOX-PCL-PEG-ColIV nanoparticles showed distributed in some organs in Figure 6 A and B, no obvious toxicity resulted from the treatment because the nanoparticles can be accumulated in the capillary basement membranes of various organs but hardly penetrate the capillary wall through the enhanced permeability and retention effect 48, 49.

The appearance of tumor slices under different magnifications is shown in Figure 8B. In the NS group, MCF-7 cells were intensive and overlapped. The nuclei of MCF-7 cells were identical in size and clearly visible and consisted of abundant eosinophilic granules. In the DOX-PCL-PEG-COOH nanoparticle group, tumor cell density decreased, the cells were broken, and some of the areas were vacuolated. In the DOX-PCL-PEG-ColIV nanoparticle group, the edge of the tumor tissue was intact, and ring-like vacuoles and caseous necrosis were observed. In the DOX-PCL-PEG-ColIV/CLU nanoparticle group, caseous necrosis and a large number of vacuoles in the tumor site were observed. In addition, the vacuoles gradually extended to the inside of the tumor tissue. Referring to relevant references, the structure of vacuoles was similar to that of adipocytes, and adipocytes appeared in the tumor site, which might be related to the transformation of MCF-7 tumor cells. Gerhard Christofori's team used combination therapy of MEK inhibitor (Trametinib) and diabetes medicine (Rosiglitazone) to treat breast cancer. By inhibiting the MEK function and activating the epithelial-mesenchymal retrograde transformation, breast cancer cells can be transformed into adipocytes and maintain adipocyte morphology, which can effectively inhibit the invasion of cancer cells 50. These studies suggested that DOX-PCL-PEG-ColIV/CLU nanoparticles may stimulate epithelial-mesenchymal retrograde transformation, thereby leading to the transformation of tumor cells into adipocytes, and caseous necrosis may be associated with DOX delivered to the tumor tissue.

**Figure 9:**
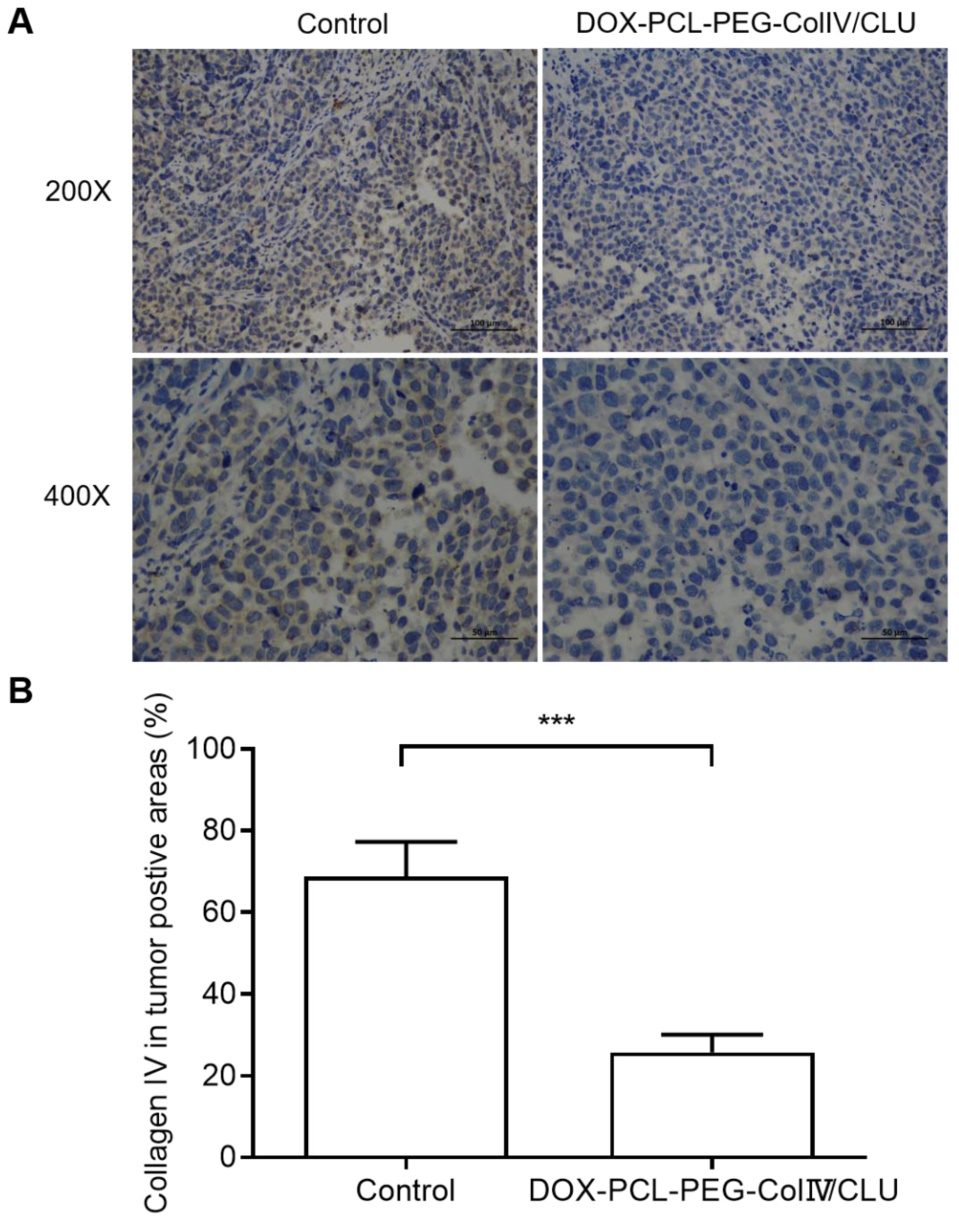
Collagen IV levels in tumor tissues before and after treated with DOX-PCL-PEG-ColIV/CLU nanoparticles. (A) HE staining (200× and 400×). (B) Percentage of collagen IV positive cells, ^***^p < 0.001.

In order to clarify the degradation effect of DOX-PCL-PEG-ColIV/CLU on collagen IV, the collagen IV levels in tumor tissues were also compared between control and nanoparticle groups after given the preparations to mice every 2 days for 10 consecutive days. As shown in Figure 9, DOX-PCL-PEG-ColIV/CLU could degrade the collagen IV protein *in vivo* condition, showing significantly lower collagen IV positive cells than control group. This also indicates that the collagenase IV-modified nanoparticles can work *in vivo* for the degradation of collagen IV and penetrate the dense tumor tissues.

## Conclusions

In this study, we designed novel PCL-PEG nanoparticles modified with ColIV and CLU. These nanoparticles load DOX to increase the penetration to the tumor ECM and reduce the phagocytosis by the reticuloendothelial system. The particle size of DOX-PCL-PEG-ColIV/CLU nanoparticles was 151.4 nm, and they had regular spherical structure and relatively high encapsulation efficiency. DOX-PCL-PEG-ColIV/CLU nanoparticles exhibited a distinct reduction of phagocytosis in RAW264.7 cells and a significant improvement of penetration to tumor ECM, which was confirmed by the 2D and 3D ECM models. In addition, the result of tissue distribution strongly suggested that DOX-PCL-PEG-ColIV/CLU nanoparticles significantly reduced the accumulation in the liver and kidney. The nanoparticles combined with ColIV and CLU can well exert antitumor ability. DOX-PCL-PEG-ColIV nanoparticles may stimulate epithelial-mesenchymal retrograde transformation, thereby leading to the transformation of tumor cells into adipocytes. Caseous necrosis may be associated with DOX delivered to the tumor tissue. DOX-PCL-PEG-ColIV/CLU nanoparticles showed high safety, excellent cellular uptake in MCF-7 cells, and penetration ability through the ECM, antiphagocytosis ability, and antitumor effects both *in vivo* and *in vitro*. This study provided a theoretical basis for investigating long circulating nanocarriers to overcome the tumor ECM barriers. Because DOX-PCL-PEG-ColIV/CLU nanoparticles can degrade not only collagen in the tumor tissue but also the collagen matrix in the normal tissues including blood vessels, more detailed study should be conducted on potential toxicity issues for further applications.

## Supplementary Material

Supplementary figures, tables, methods, results.
