# Supplementary material for: Collagenase IV and clusterin-modified polycaprolactone-polyethylene glycol nanoparticles for penetrating dense tumor tissues
Source: Theranostics. 2021 Jan 1;11(2):906–24. doi: 10.7150/thno.47446 (PMC7738847; doi:10.7150/thno.47446)
Supplement: Supplementary file 1 — Supplementary figures, tables, methods, results. [file thnov11p0906s1.pdf]

Supporting Information

Collagenase IV and clusterin-modified polycaprolactone-polyethylene glycol nanoparticles for penetrating dense tumor tissues

Hao-Yan Huang[a,1], Li-Qing Chen[a,1], Wei Sun[a], Huan-Huan Du[a], Shunli Dong[a], Atef Mohammed Qasem Ahmed[a], Dingyun Cao[b], Jing-Hao Cui[a], Yi Zhang[a,#], Qing-Ri Cao[a,*]

[a]College of Pharmaceutical Sciences, Soochow University, Jiangsu Province, Suzhou 215123, People's Republic of China
[b]Suzhou No3 high school sino-us course center, Jiangsu Province, Suzhou 215001, People's Republic of China

[*]Qing-Ri Cao, E-mail: qrcao@suda.edu.cn, Tel: 86-138-62012952.
[#]Yi Zhang, E-mail: Zhangyi@suda.edu.cn, Tel: 86-512-65208583.

[1] These authors contributed equally to this work.

Methods

Synthesis of PCL-PEG-ColIV

The method has been already described in the main text.

Differential scanning calorimetry (DSC)

To further investigate whether PCL-PEG-ColIV was synthesized, the thermal behavior of PCL-PEG-COOH and PCL-PEG-ColIV was measured using DSC. Each sample of 2 mg was placed in a sealed pan and heated from 25 °C to 500 °C at a scanning rate of 20 °C/min under a nitrogen flow of 40 mL/min.

Critical micelle concentration (CMC)

Pyrene standard solution (10 μg/mL) of 25 μL was placed in each glass bottle and heated at 40 °C for 0.5 h for the evaporation of organic solvent. The micelle solutions with different concentrations were diluted and injected into glass bottles, wherein pyrene concentration was 6×10^{-7} mol/L. PCL-PEG-COOH micelles of 1 mg/mL were diluted to the concentrations of 1×10^{-5}, 5×10^{-5}, 1×10^{-4}, 5×10^{-4}, 1×10^{-3}, 2.5×10^{-3}, 5×10^{-3}, 1×10^{-2}, and 0.5 mg/mL, whereas 1 mg/mL of ColIV-modified micelles was diluted concentrations of 1×10^{-5}, 1×10^{-4}, 2.5×10^{-3}, 5×10^{-3}, 1×10^{-2}, 2.5×10^{-2}, 0.1, 0.5, and 1 mg/mL. The mixed solution was ultrasonicated using an ultrasound probe for 0.5 h and incubated at 40 °C for 1 h and stored for up to 12 h at room temperature away from light. Each sample solution of 1 mL was determined with excitation wavelength of 334 nm, excitation width slit of 5.0 nm, emission wavelength from 350 nm to 450 nm, emission wavelength slit of 2.5 nm, and scanning speed of 50 nm/min.

Calibration curves

Grafting rate of ColIV

ColIV powder of 10 mg was weighed and placed in a 10 mL volumetric flask and then diluted with water to obtain 1 mg/mL of ColIV standard solution. ColIV standard solution of 1, 5, 10, 20, 30, and 40 μL was added into a 96-well plate. Then, 150 μL of coomassie

brilliant blue was added and diluted to 200 μL of total volume with purified water. PCL-PEG-ColIV nanoparticles (1 mg/mL, 50 μL) were mixed with 150 μL of coomassie brilliant blue as sample solution. The OD595 absorbance was determined using enzyme micro-plate reader, and the standard curve of ColIV was drawn. The ColIV grafting rate was calculated according to the following formula:

$$\text{Grafting rate } (\%) = \frac{\text{concentration of sample} \times \text{volume of sample}}{\text{theoretical weight of Col[illegible]}} \times 100\%.$$

Enzyme activity of ColIV in PCL-PEG-ColIV nanoparticles

Calibration curves of glycine

Glycine powder at 75.07 mg was precisely weighed and placed in a 100 mL volumetric flask. Glycine solution at 0.3 mmol/L was obtained by the dilution with purified water. Ninhydrin powder at 1.5 g was also weighed and placed in 100 mL of volumetric flask by dissolving with PBS solution (pH 5.4). Glycine solutions at 0.1, 0.4, 0.5, 0.6, and 0.8 mL were added into tubes and then diluted with 1 mL of purified water. A series of glycine solution was mixed 1 mL of PBS solution (pH 5.4) and 1 mL of ninhydrin solution. The resulting mixture was used to calibrate the standard solution of glycine to determine the enzyme activity of samples.

Preparation of sample

The water-soluble amino acids and short peptides of collagen hydrolysate were determined by ninhydrin colorimetry. PCL-PEG-ColIV nanoparticles of 1 mg/mL were prepared as sample solution, and 3.0 mL of ColIV solution (1 mg/mL) was transferred into 100 mL of volumetric flask and diluted with pure water as control solution. Thus, the enzyme concentration of the sample solution was equal to that of the control solution. Sample solution and control solution were used as enzyme solution to participate in the reaction system. The reaction system consisted of 0.6 mL gelatin solution (0.24%, w/w), 0.4 mL PBS buffer solution (pH 7.4), and 0.2 mL enzyme solution, which were reacted at 37 °C for 30 min and then cooled down to room temperature as sample solution. The sample solution was mixed with 1 mL of pH 5.4 PBS solution at 1 mL of ninhydrin solution. The two reaction systems above were heated in boiling water for 20 min, cooled for 5 min in cold water, and then mixed with 3 mL ethanol (60%, v/v) to prevent precipitation, which affected the absorbance determination. After shaking, 200 μL of the solution was added into a 96-well plate, and the OD570 absorbance was measured by an enzyme microplate reader. The standard curve of glycine was drawn, and enzyme activity of ColIV in PCL-PEG-ColIV nanoparticles was determined.

Calibration curve of DOX

The fluorescence values of 0, 0.01, 0.025, 0.05, 0.1, 0.25, 0.5, 1, and 5 μg/mL DOX standard solutions were determined, and the calibration curve was drawn. DOX, DOX-PCL-PEG-COOH, DOX-PCL-PEG-ColIV, and DOX-PCL-PEG-ColIV/CLU nanoparticles (1 mg of DOX) of 5 mL were transferred to dialysis bags (3500 MW) and suspended in 1000 mL purified water for 24 h. The fluorescence values were measured with 2 mL dialysis solution. The encapsulation efficiency and drug loading of nanoparticles were calculated according to the following formulas:

$$\text{Drug loading } (\%) = \frac{\text{weight of total DOX} - \text{weight of free DOX}}{\text{weight of carrier}} \times 100\%$$

$$\text{Encapsulation efficiency } (\%) = \frac{\text{weight of total DOX} - \text{weight of free DOX}}{\text{weight of total DOX}} \times 100\%.$$

Calibration curve of BSA

BSA is commonly used as a standard substance in the determination of plasma protein concentration, whereas CLU is one of the components of plasma protein. Therefore, this experiment selected BSA to establish a standard curve to determine the CLU concentration.

BSA powder of 10 mg was weighed and placed in a 10 mL volumetric flask to obtain 1 mg/mL BSA standard solution by diluting with purified water. BSA standard solutions of 2, 4, 6, 8, and 10 μL were added into a 96-well plate. Then, the solution was diluted with purified water to 20 μL, followed by adding 180 μL coomassie brilliant blue solution. The supernatant of 10 mg/mL DOX-PCL-PEG-ColIV/CLU nanoparticle solution was obtained after centrifugation at 10,000 rpm for 30 min. The sample solution at 10 μL was also added to a 96-well plate and processed in the same manner as the BSA standard solution. The OD595 absorbance was measured by an enzyme microplate reader within 30 min. The standard curve of BSA was drawn, and the concentration of free CLU was calculated according to the OD595 value of the sample.

Characterization of PCL-PEG-ColIV/CLU nanoparticles

PCL-PEG-COOH, PCL-PEG-ColIV, and PCL-PEG-ColIV/CLU nanoparticles (0.2 mg/mL polymer) were prepared and dispersed in distilled water by ultrasound for 30 min. The particle size and zeta potential of the samples were measured using a Zetasizer (HPP 5001, Malvern. UK). Three different nanoparticles were dripped on copper meshes coated with a carbon support film and then dried under infrared lamp. The morphology of nanoparticles was observed by TEM (120 kV, HT-7700, Hitachi, Japan).

FT-IR

To further investigate whether PCL-PEG-COOH and PCL-PEG-ColIV nanoparticles were successfully prepared, their spectra were recorded using an FT-IR spectroscope at a wavelength range of 500–4000 cm^{-1}.

2D and 3D ECM models

The constituents in Table S1 were mixed in ice bath, and the bubbles were removed after 1 h. The above mixture was added to the head of quartz capillary (0.5 mm inside diameter, 0.35 mm external diameter, and 80 mm length) with a syringe. The head and end were sealed with a sealing film and placed in the water bath at 37 °C for 12 h.

Table S1. Components of the 2D ECM Model

Composition	Volume (μL)
10×PBS	48
NaOH solution (1N)	12.8
Purified water	9.2
HA solution (5 mg/mL)	80
Collagen type I of rat tail solution (5 mg/mL)	320
Gelatin coating solution (0.24%, w/w)	330
Total volume	800

The 3D ECM model was established by screening different proportions of chitosan solution and collagen coating solution, as shown in Table S2. Chitosan solution (cht, 0.24% w/w) and collagen solution (col, 0.24% w/w) were mixed in an ice bath at six different

proportions, namely, 9:1, 3:1, 1:1, 1:3, 1:9, and 0:1. In addition, 1 N NaOH solution was added to adjust the pH to neutral. After 2 h of ultraviolet sterilization, the mixture was placed at 37 °C and 5% CO_2 cell incubator for 30 min. MCF-7 cells were digested with a count of 2×105 cells/mL. Then, 2 mL of the cellular suspension was incubated with gel at each well for 24 h. The 3D ECM model was observed and photographed by using an optical microscope.

Table S2. 3D ECM model with different chitosan/collagen ratios

Solutions	V_{cht} (μL)	V_{col} (μL)	V_{NaOH} (μL)
Cht/Col-10	900	100	162
Cht/Col-25	750	250	130
Cht/Col-50	500	500	95
Cht/Col-75	250	750	55
Cht/Col-90	100	900	33
Col	0	1000	15

Penetration effects of ColIV amount in the 2D ECM model

To further confirm the ColIV (10 U) modification on the penetration ability of nanoparticles, the penetration of nanoparticles physically mixed with different amounts of ColIV (10 U, 100 U or 1000 U) in the 2D ECM model were studied.

Tissue fluorescence distribution at 12 h

This study investigated the tissue fluorescence distribution of nude mice after administration of DOX-PCL-PEG-COOH nanoparticle and DOX-PCL-PEG-ColIV nanoparticle at 12 h.

Results

Synthesis of PCL-PEG-ColIV

As shown in Figure S1A, PCL-PEG-ColIV was synthesized by a carbodiimide method. The brown powder of PCL-PEG-ColIV was formed and stored at −20 °C (Figure S1B).

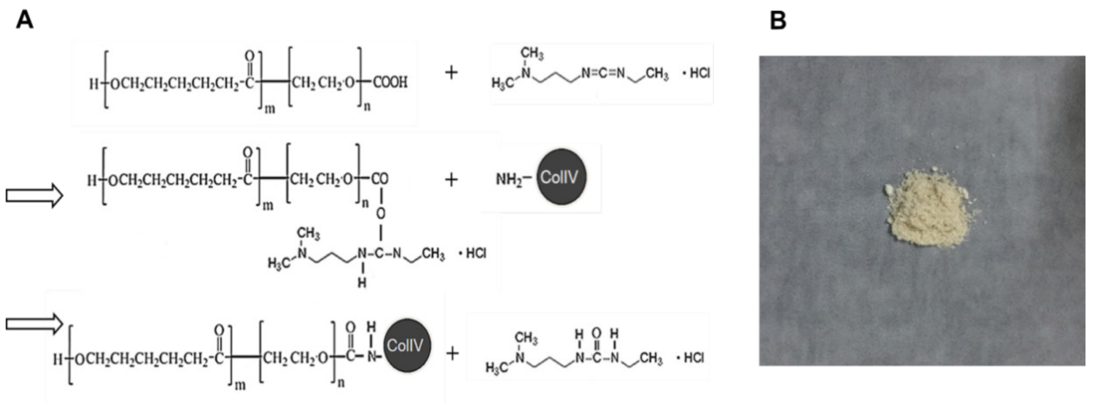

Figure S1. Synthesis of PCL-PEG-ColIV. A) Scheme of synthesis; B) Powder sample of PCL-PEG-ColIV.

DSC

The melting point of the endothermic peak of PCL-PEG-COOH was 333 °C, whereas that of PCL-PEG-ColIV was 374 °C as shown in Figure S2. The significant change of melting point meant that ColIV-modified PCL-PEG (PCL-PEG-ColIV) had been successfully synthesized.

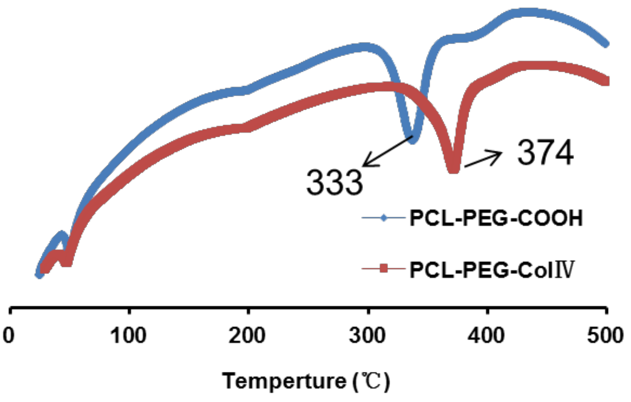

Figure S2. DSC spectra of PCL-PEG-COOH (blue line) and PCL-PEG-ColIV (red line).

CMC

As shown in Figure S3, the CMC of PCL-PEG-COOH and PCL-PEG-ColIV was 1.5 and 15 μg/mL, respectively. The CMC increase showed that the molecular weight of hydrophilic chain increased, indicating the PCL-PEG-ColIV formation.

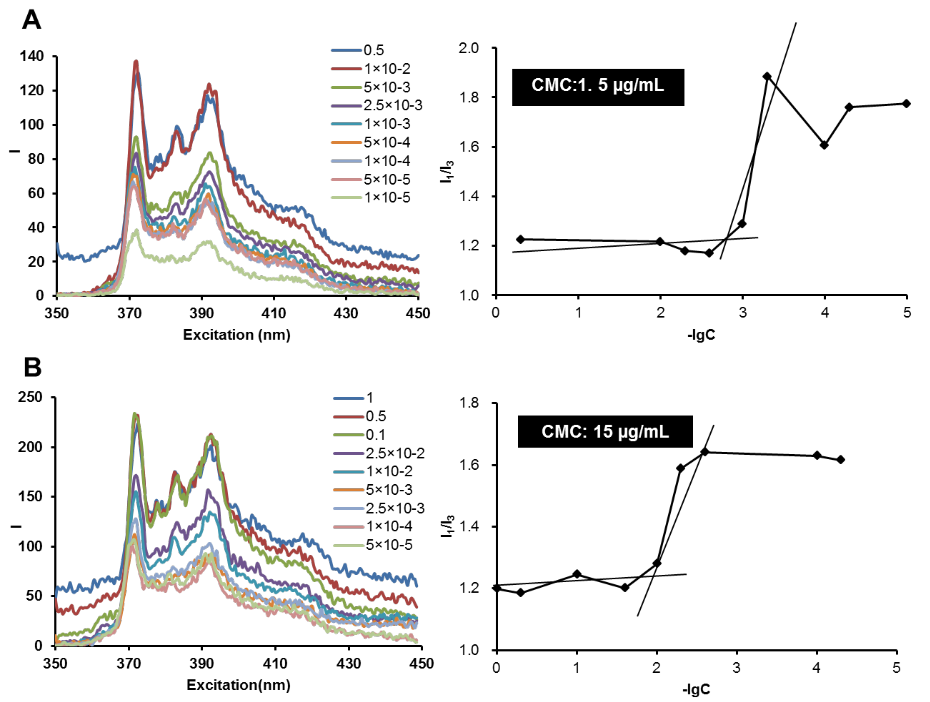

Figure S3. Full wavelength fluorescence spectra of pyrene in micelle solution for CMC (A) PCL-PEG-COOH; (B) PCL-PEG-ColIV.

Calibration curves

Grafting rate of ColIV

As calculated, 1 mg of PCL-PEG-ColIV consisted of 58.86 μg of ColIV. Theoretically, 61.03 μg of ColIV in 1 mg of PCL-PEG-ColIV was observed. Thus, the grafting rate of ColIV was 96.44%.

Enzyme activity of ColIV in PCL-PEG-ColIV nanoparticles

As shown in Figure S4B**,** the calibration curve of glycine showed a good linearity at the concentration range of 0.1–0.8 mL of glycine (R^2=0.9993). The ColIV activity in PCL-PEG-ColIV nanoparticle solution was calculated to be 8.53 U, and that in the ColIV solution containing the same amount of enzyme was 8.55 U. Therefore, the activity of ColIV-modified nanoparticles was 99.74%. There was no significant loss of ColIV activity during PCL-PEG-ColIV synthesis and nanoparticle preparation.

Standard curve of DOX

As shown in Figure S4C, the calibration curve of DOX showed a good linearity at the concentration range of 1–5 μg/mL (R^2=0.9952). The three nanoparticles prepared by solvent evaporation had high encapsulation efficiencies.

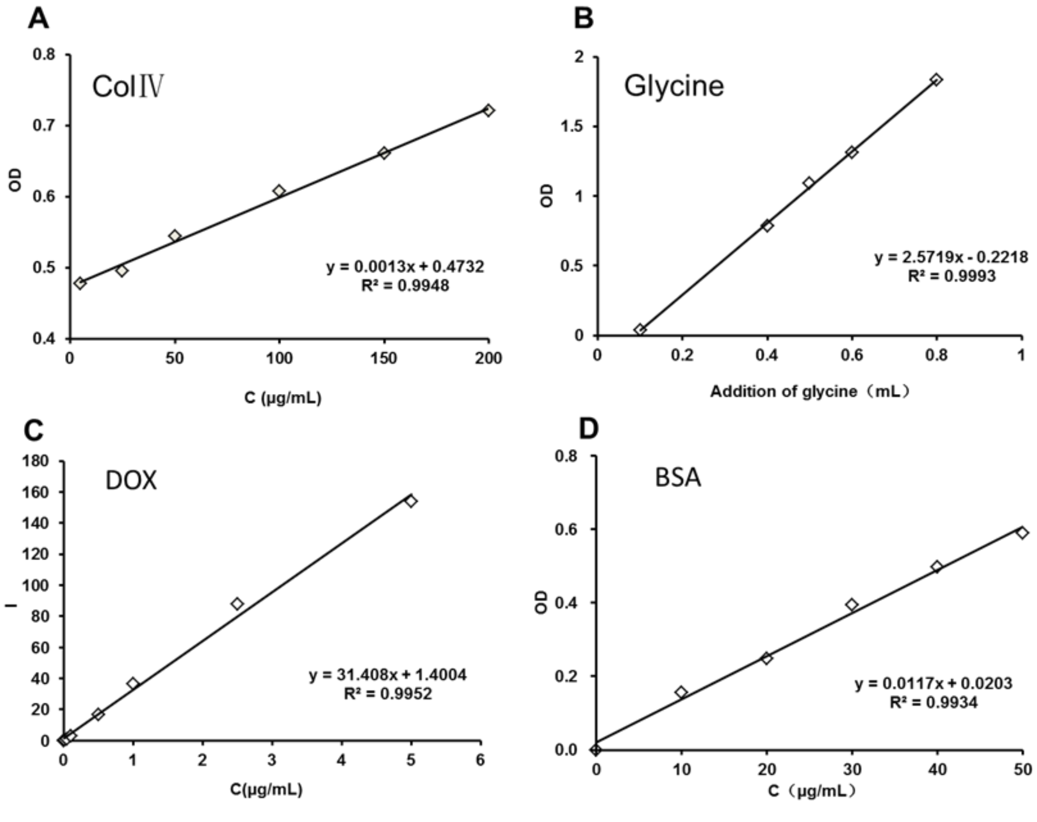

Figure S4. Four calibration curves of ColIV, glycine, DOX, and BSA. (A) ColIV. The grafting rate of ColIV-linked to PCL-PEG-COOH can be detected. (B) Glycine. Glycine was determined by ninhydrin colorimetry as standard amino acid, and degradation product of gelatin with ColIV solution and PCL-PEG-ColIV nanoparticle solution was detected, and the activity of ColIV-modified nanoparticles was calculated. (C) DOX. DOX standard solution determined and the standard curve was drawn. The encapsulation efficiency and drug loading of nanoparticles were calculated. (D) BSA. BSA was used as a standard substance in the determination of concentration of free CLU.

Standard curve of BSA

As shown in Figure S4D, the calibration curve of BSA showed a good linearity in the concentration range of 0–50 µg/mL. The concentration of free CLU from the centrifuged supernatant was 1.55 g/mL, and total CLU concentration in 10 mg/mL of DOX-PCL-PEG-ColIV/CLU nanoparticle solution was 10 µg/mL. Thus, the calculated adsorption rate of CLU was 84.54%. CLU was efficiently modified onto DOX-PCL-PEG-ColIV nanoparticles.

Characterization of PCL-PEG-ColIV/CLU nanoparticles

The appearances of all three blank nanoparticle solutions were clear and transparent, as shown in Figure S5A. The particle size and zeta potential of PCL-PEG-COOH, PCL-PEG-ColIV, and PCL-PEG-ColIV/CLU nanoparticles were measured by a laser particle size analyzer in Figure S5B. After being modified with ColIV, the particle size of DOX-PCL-PEG-ColIV nanoparticles increased from 68.0 nm to 150.4 nm, which was related to the increase of molecular weight of enzymes. After further adsorption with CLU, the particle size was 151.8 nm. As shown in Figure 5C**,** the morphology of nanoparticles was further confirmed by TEM. PCL-PEG-COOH nanoparticles, PCL-PEG-ColIV, and PCL-PEG-ColIV/CLU nanoparticles had regular spherical structures.

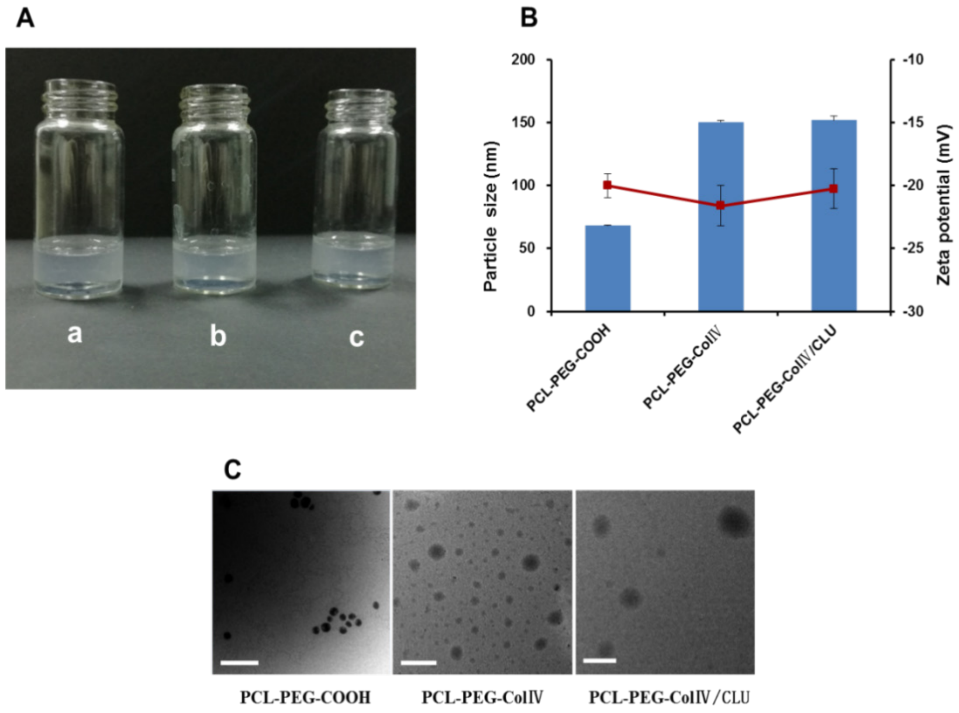

Figure S5. Three types of nanoparticle solutions (a: PCL-PEG-COOH; b: PCL-PEG-ColIV; c: PCL-PEG-ColIV/CLU). (A) Appearance. (B) Particle size and zeta potential. (C) TEM images.

FT-IR

As shown in Figure S6A, C=C double bond of DOX in the range of 1620−1450 cm^{-1} disappeared in the DOX-PCL-PEG-COOH nanoparticle group, thereby proving that DOX had been encapsulated into PCL-PEG-COOH nanoparticles. As shown in Figure S6B, new absorption peaks at 1589.26 cm^{-1} appeared in the DOX-PCL-PEG-ColIV nanoparticle group, which also proved that DOX reacted with PCL-PEG-ColIV nanoparticles.

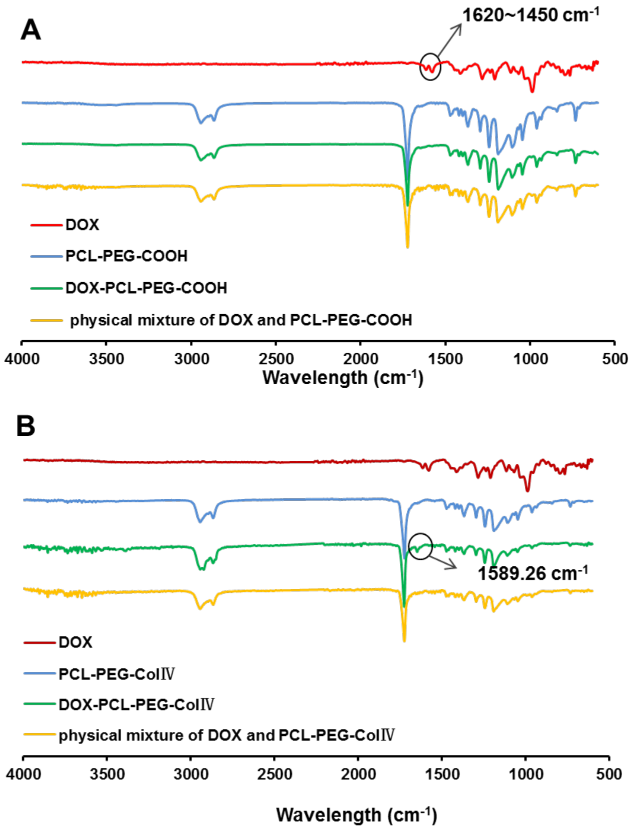

Figure S6. FT-IR spectra. (A) PCL-PEG-COOH. (B) PCL-PEG-ColIV/CLU.

2D and 3D ECM models

After gel was formed in a quartz capillary tube (Figure S7A), the 2D ECM model was successfully established. Most MCF-7 cells were suspended in the gel of the ECM 3D model and clustered agglomeration in the ratio of cht and col at the ratio of 1 to 3 (Figure S7B). By contrast, the appearance of MCF-7 cells cultured on 2D plates was an adhere-wall. The significant differences in morphological structure meant that 3D ECM model could better simulate the living environment of tumor cells *in vivo*.

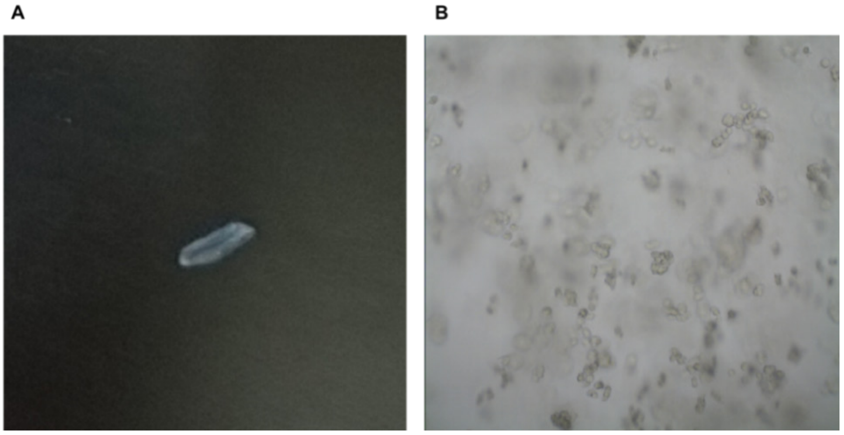

Figure S7. ECM 2D and 3D models. (A) Gel formation of the 2D ECM model, (B) Appearance of ECM 3D model.

Penetration effects of ColIV amount in the 2D ECM model

As shown in Figure S8, DOX-PCL-PEG-COOH nanoparticles physically mixed with the amount of effective ColIV (10 U 100 U) penetrated to the middle of 2D ECM gel in the capillary. Meanwhile, nanoparticles physically mixed with 1000 U of ColIV penetrated the terminal part of the capillary.

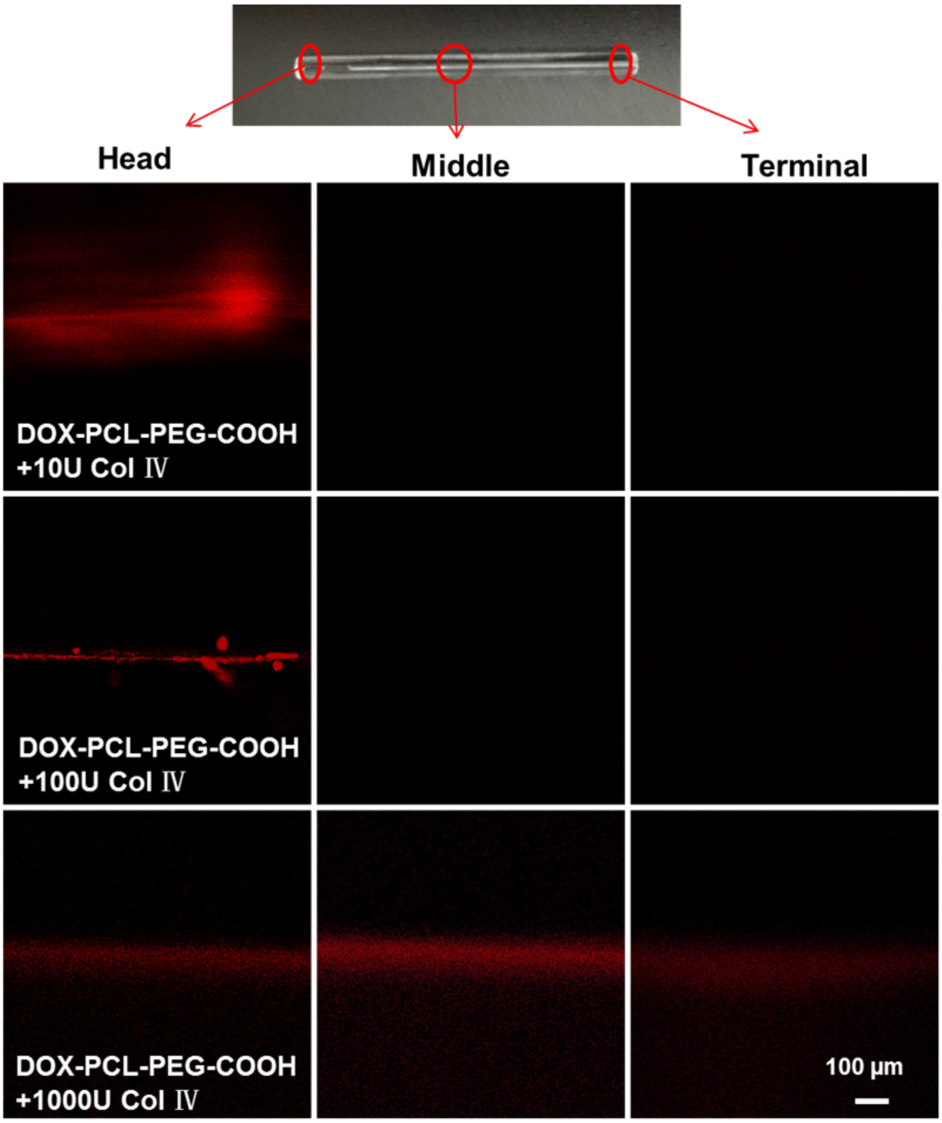

Figure S8. Penetration effects of ColIV amount in the 2D ECM model.

Tissue fluorescence distribution at 12 h

As shown in Figure S9, DOX-PCL-PEG-COOH nanoparticles showed fluorescence, but the DOX-PCL-PEG-ColIV nanoparticle's fluorescence in the tumor tissue was less. DOX-PCL-PEG-COOH nanoparticles experienced less phagocytosis caused by the PEG chain on the surface at 12 h, thereby resulting in increased uptake at the tumor site. However, due to the good penetration of ColIV, DOX-PCL-PEG-ColIV nanoparticles penetrate the visceral tissues rich in collagen at 12 h, thereby resulting in a remarkable decrease in the uptake of DOX-PCL-PEG-ColIV nanoparticles at the tumor site. Meanwhile, the fluorescence

intensity of DOX-PCL-PEG-COOH nanoparticles decreased with the growth of tumors at 72 h, whereas DOX-PCL-PEG-ColIV nanoparticles redistributed from other organs to tumors, thereby resulting in significant tumor accumulation. The main reason for the lower antitumor effect of DOX-PCL-PEG-ColIV nanoparticles compared with that of DOX-PCL-PEG-COOH nanoparticle was that the dynamic equilibrium of distribution in different kinds of nanoparticles differed *in vivo*.

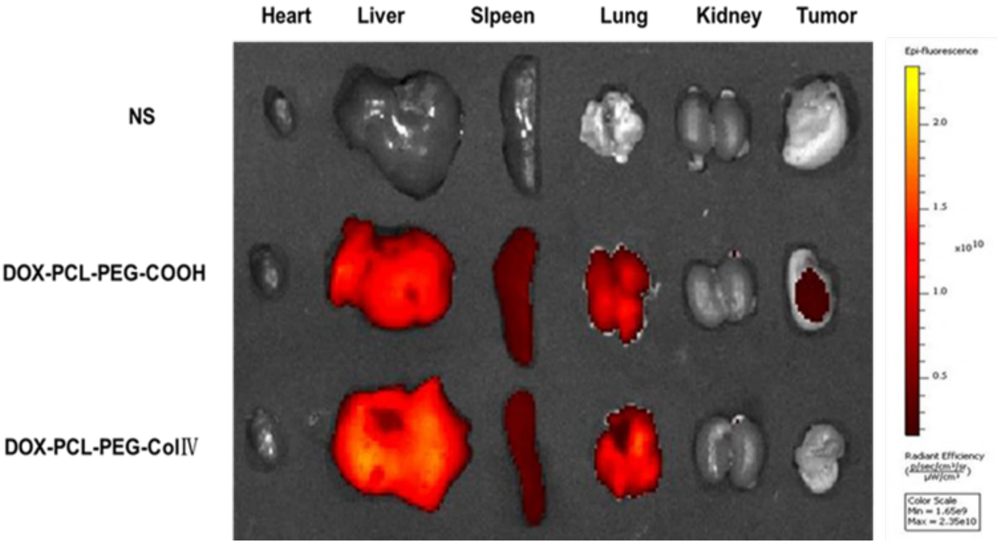

Figure S9. Fluorescence images of various organs after administration of nanoparticles through the tail vein at 12 h.
